# Tree cover loss and intensified land use simplify bat assemblages in Amazonian cacao agroforestry landscapes

**DOI:** 10.1007/s10980-026-02371-6

**Published:** 2026-05-06

**Authors:** Pablo Aycart-Lazo, Luz Sánchez-Maldonado, Blanca Ivañez-Ballesteros, Carolina Ocampo-Ariza, Stefan Dullinger, Evert Thomas, Teja Tscharntke, Ingolf Steffan-Dewenter, Bea Maas

**Affiliations:** 1 Department of Botany and Biodiversity Research, University of Vienna, Vienna, Austria; 2 Department of Animal Ecology and Tropical Biology, University of Würzburg, BiocenterWürzburg, Germany; 3 División de Ecología Vegetal, Centro de Ornitología y Biodiversidad (CORBIDI), Lima, Peru; 4 Facultad de Ciencias, Universidad Nacional Agraria La Molina, Lima, Peru; 5 Department of Crop Sciences, Functional Agrobiodiversity and Agroecology, University of Göttingen, Göttingen, Germany; 6 Bioversity International, Lima Office, Lima, Peru; 7 Ecological Networks, Technische Universität Darmstadt, Darmstadt, Germany; 8 Department for Integrative Biology and Biodiversity Research, Institute of Zoology, BOKU University, Vienna, Austria

**Keywords:** Aerial insectivorous bats, Agricultural deforestation, Biotic homogenization, Peru, Phyllostomidae, Phylogenetic diversity

## Abstract

**Context:**

Agricultural expansion threatens biodiversity in Amazonian landscapes. Cacao agroforests can support multifunctional bat assemblages, which are sensitive to landscape-level agricultural intensification and may provide information for the development of land-use policies in the Amazon.

**Objectives:**

We assessed how landscape structure around cacao agroforests affected aerial insectivorous bats and phyllostomid bats (mainly frugivores) and how cropland expansion or reforestation may affect future bat diversity.

**Methods:**

We recorded aerial insectivores and captured phyllostomids in 28 cacao agroforests in two neighboring regions in the Peruvian Amazon that differ in the proportion of remaining forest and intensified cropland, and analyzed their taxonomic, functional and phylogenetic responses to current and future landscape structure.

**Results:**

Aerial insectivore activity (an abundance surrogate) and feeding buzzes (a foraging activity indicator) decreased with increasing cropland cover in the non-intensive region only, highlighting the higher vulnerability of these assemblages to cropland expansion. Aerial insectivore phylogenetic diversity, but not functional diversity, decreased with landscape tree cover loss in the non-intensive region. Lower tree cover resulted in higher abundances of dominant frugivores in both regions, while higher edge density was associated with more insect-eating phyllostomids. Frugivore abundance decreased with increasing cropland cover in the intensive region. Our results predicted fewer feeding buzzes and insect-eating phyllsotomids under ongoing deforestation, potentially impacting bat-mediated pest control. Deforestation may also reduce frugivore Simpson diversity in the non-intensive region.

**Conclusions:**

Limiting monoculture expansion and restoring landscape tree cover could mitigate taxonomic and phylogenetic homogenization of bat assemblages and the loss of potential ecosystem functions in Amazonian agricultural areas.

**Supplementary Information:**

The online version contains supplementary material available at 10.1007/s10980-026-02371-6.

## Introduction

Bat diversity peaks in the Amazon basin, with roughly 10% of the 1,500 known bat species occurring there, which is reflected in the wide diversity of body sizes, diets and flight strategies of Amazonian bats (Lopez-Baucells et al. 2016). This high diversity makes Amazonian bat assemblages particularly vulnerable to the increasing threats from both the expansion and intensification of agriculture in the region, but also valuable ecological indicators and relevant ecosystem service providers (Albert et al. 2023; Kunz et al. 2011; Medellín et al. 2000). Forest conversion into agricultural land can also have negative consequences for crop yield due to the reduction of ecosystem service providers, such as pollinators and pest controllers (Dainese et al. 2019). However, low-intensity land use can help mitigate defaunation in tropical agricultural landscapes. By integrating shade trees into cacao plantations, cacao agroforests can provide refuge and resources for biodiversity not found in more intensive systems, such as cacao monocultures, enhancing the long-term sustainability of food production and the stability of biological communities in these landscapes (Farneda et al. 2020; Tscharntke et al. 2011).

Amazonian bat assemblages are highly diverse, with up to nine different bat families living in sympatry in a single site (Lopez-Baucells et al. 2016). Of these, the most speciose and functionally diverse family, and the only one including phytophagous species, is Phyllostomidae (Neotropical leaf-nosed bats), which includes 106 of the 187 bat species found in Peru (Pacheco et al. 2021). This family comprises mainly frugivorous species, but also species that feed on nectar, pollen, arthropods, vertebrates or blood (Rojas et al. 2011). Phyllostomids rely more on sight and smell than on echolocation for resource acquisition and have various sizes of up to ca. 180 g (Korine & Kalko 2005). The remaining families comprise only aerial-hawking insectivorous bats (hereafter ‘aerial insectivores’), which usually have small body sizes and use echolocation to hunt flying insects in the air (Jones 1999). These highly divergent feeding preferences and strategies make Amazonian bats important providers of ecosystem services. Together with birds, insectivorous bats can reduce phytophagous arthropod abundance in Neotropical agroforestry systems (Cassano et al. 2016; Ocampo-Ariza et al. 2023; Schmitt et al. 2021). Further, frugivorous bats are among the most important dispersers of pioneer trees (e.g., *Cecropia* or *Vismia* species) in the Amazon, contributing to the regeneration of abandoned agricultural lands (Muscarella & Fleming 2007). Therefore, studying both insectivorous and frugivorous bats simultaneously is necessary to gain a full perspective of the Amazonian bat community and the impacts of land-use change on its diversity and ecosystem services (Kalko et al. 1996).

Agricultural practices and land-use change have been found to impact bat guilds differently, usually leading to higher functional and phylogenetic homogeneity of bat assemblages in simplified Neotropical landscapes (Farneda et al. 2020, 2024; Frank et al. 2017). Frugivorous phyllostomids tend to become more dominant in fragmented landscapes with high abundance of pioneer plants on which they feed (Gorresen & Willig 2004; Klingbeil & Willig 2009), while insectivorous phyllostomids usually benefit from closed canopy forests and higher landscape-level edge density (Chambers et al. 2016; Klingbeil & Willig 2009). The responses of aerial insectivores to landscape composition and configuration can also vary depending on their dispersal capacity, being open-area foragers with large sizes and high wing loadings typically less affected by forest loss and fragmentation (Bader et al. 2015; Colombo et al. 2023; García-Morales et al. 2013). Moreover, the responses of bat assemblages to deforestation may also differ based on regional factors such as seasonality, type of forest cover or deforestation history (Ocampo-Ariza et al. 2024; Socolar et al. 2019). Highly seasonal climates, typical of Amazonian dry forests, and longer deforestation histories filter regional species pools by selecting functional traits associated with higher tolerance to disturbances. This leads to species assemblages adapted to changing environments, which typically respond less strongly to current land-use changes (Hua et al. 2024). Understanding and accounting for these regional variations in bat responses to agricultural intensification would likely improve the outcomes of biodiversity-friendly landscape management policies in tropical regions (Tscharntke et al. 2005).

Despite the importance of the Amazon basin for bat conservation, most studies on Neotropical bats in agricultural landscapes have been conducted in Central America or the Brazilian Atlantic forest (Farneda et al. 2020; Xavier et al. 2023). Additionally, previous studies mainly focused on phyllostomids, with only 25% of the studies targeting aerial insectivores (Xavier et al. 2023) and just a few sampling both groups (e.g., Heer et al. 2015; Pereira et al. 2018). Considering the current rates of deforestation and land-use change in the Amazon (Albert et al. 2023), it is essential to understand how these trends may shape future bat assemblages and inform the development of landscape management strategies aimed at enhancing bat conservation and ecosystem services (Brasileiro et al. 2022).

In this study, we assessed how the taxonomic, functional and phylogenetic diversity and potential ecosystem functions of aerial insectivore and phyllostomid assemblages in cacao agroforests varied depending on the surrounding landscape structure in the Peruvian Amazon. Specifically, we assessed bat responses to cropland and tree cover, as well as edge density, as proxies of agricultural intensification and fragmentation in the surroundings of the cacao agroforests (Fahrig 2003). To analyze whether the regional context influenced bat responses, we conducted the assessments in two nearby regions: one dominated by industrial rice and papaya monocultures (intensive region) and another by smallholder systems, including cacao agroforests and banana plantations (non-intensive region). Furthermore, we explored how future trends of bat communities might shift in each region by projecting their responses to two opposed landscape scenarios forecasting tree cover loss or gain due to cropland expansion or reforestation, respectively. This study combines functional and phylogenetic metrics with projections of potential ecosystem functions under future land-use scenarios, a needed approach for conservation planning in tropical agriculture (Brasileiro et al. 2022; Gonçalves et al. 2021).

We hypothesized that: (1) surrounding land-use intensity will have negative effects on the diversity and abundance of bat assemblages in cacao agroforests of the non-intensive region, which are expected to have a high proportion of disturbance-sensitive species. On the other hand, we expect bat assemblages in the intensive region to be primarily composed of disturbance-tolerant species and therefore show attenuated responses to the surrounding landscape composition and configuration. (2) Land-use intensification effects are guild-dependent. Specifically, we hypothesized that the richness and abundance of frugivorous phyllostomids would be higher in agroforests surrounded by high tree cover and edge density due to greater availability of pioneer plants and food resources in these landscapes. Conversely, given their high reliance on closed canopy forests, insectivorous phyllostomids and understory-foraging aerial insectivores would decline with increasing cropland cover and decreasing tree cover in the surroundings of the agroforest. Finally, (3) these guild-specific effects would lead to higher taxonomic, functional and phylogenetic homogeneity of future aerial insectivore and frugivore assemblages with potential agricultural intensification of the landscape. This homogenization would be driven by the selection of phylogenetically-related frugivorous phyllostomids and disturbance-adapted species with large sizes, high dispersal capacity and open-area foraging habits.

## Materials and methods

### Study area

**Fig. 1:**
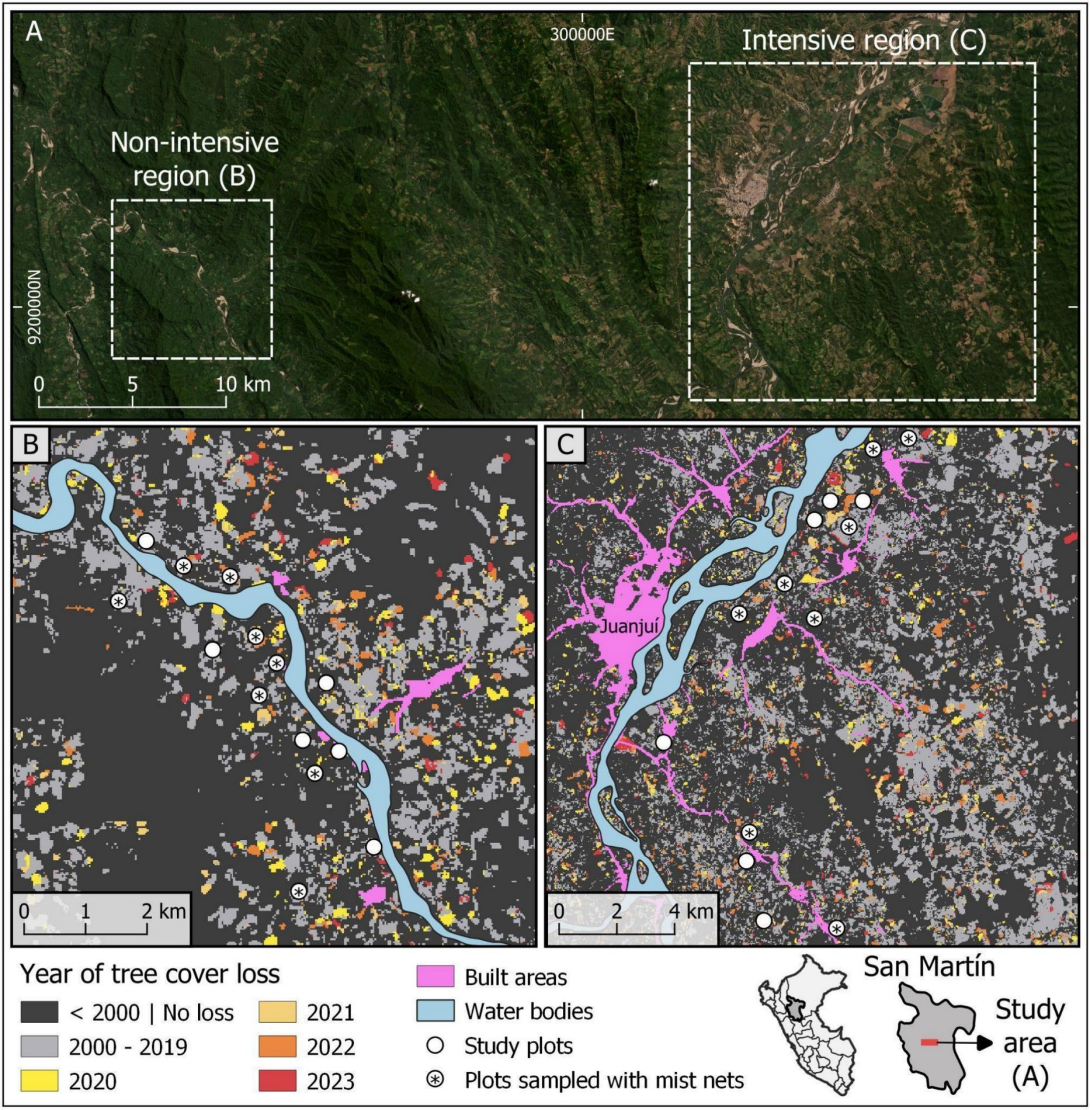
Satellite image of the study regions in the northern Peruvian Amazon (San Martín department) extracted from PlanetScope (August 2023; **A**). Distribution of the study plots and tree cover loss from 2000 to 2023 in the Huayabamba (non-intensive region; **B**) and Huallaga (intensive region; **C**) river valleys. Tree cover loss data were extracted from (Hansen et al. 2013)

We sampled aerial insectivorous and phyllostomid bats in 28 organic cacao agroforests (plots) in the San Martín department, in the northern Peruvian Amazon (Fig. 1). San Martín has the largest agricultural cover in Peru, consisting mainly of rice, banana, oil palm, coffee and cacao plantations (MIDAGRI 2021). The department has undergone significant land-use changes in recent decades, losing 11% of its primary forests between 2002 and 2023 (Global Forest Watch, www.globalforestwatch.org). The selected agroforests combined 3–4 m tall cacao trees planted in 3 × 3 m grids and shade trees of different species (mainly timber and fruit trees), maintaining an average of ca. 35% of canopy cover (± 15% SD; see Online Resource A.1 for additional information on plot characteristics).

The plots were located in two nearby regions (ca. 25 km apart from each other) chosen to represent contrasting degrees of landscape-level agricultural intensity (14 plots in each region). The Huallaga river valley (hereafter “intensive region”) has low forest cover and a predominance of intensive land-use systems such as rice and papaya monocultures. Forest cover in this region is mainly composed of secondary seasonally dry forests. Conversely, the Huayabamba river valley (hereafter “non-intensive region”) has a greater cover of forest and low-intensity land-use systems (mainly, cacao agroforests and banana plantations). Both regions have similar average annual temperatures (26 °C) and precipitation (1200–1400 mm). However, the non-intensive region has higher humidity and rainfall during the dry season, favoring the development of lowland tropical moist forests. Being closer to the city of Juanjuí (a regional economic center), the intensive region has a longer history of deforestation by industrial monocultures, while small-scale agriculture has driven deforestation in the non-intensive region in the past two decades (Fig. 1B, C). These two processes reflect the two different forms of agriculture-related deforestation in the Amazon (Finer & Novoa 2017).

Although forest types vary by region, the lack of geographic isolation between the Huallaga Valley’s seasonally dry forests and the Amazonian matrix facilitates the exchange of mobile species, especially along the river banks in which the plots were located (Stoner & Timm 2011). In fact, 100% of bat species and 97% of bird species in these seasonally dry forests are lowland Amazonian taxa (García-Villacorta 2015; Ruelas & Pacheco 2021; Vásquez Arévalo et al. 2018). Consequently, both regions offer comparable ecological contexts regarding the species pools found in their predominant vegetation types.

### Landscape structure characterization

We used two spatial scales (250 and 500 m radius buffers) and three variables (percentages of tree and cropland cover, and edge density) to characterize landscape composition and configuration around the plots. Rather than driving landscape-level effects, these small spatial scales likely influence the capacity of bat species to reach the agroforests (Aycart-Lazo et al. 2025; Gorresen et al. 2005). Due to the lack of fine-resolution and updated land-cover information for our study area, the percentage of tree cover was calculated as the average tree cover in 10 × 10 m cells using a raster layer obtained from Brandt et al. (2023). Given that a significant part of the tree cover in our study area was composed of tree orchards, banana plantations and cacao monocultures, we estimated cropland cover based on the manual classification of satellite images extracted from PlanetScope for September 2022 on QGIS v3.28.1 corrected through ground-truthing and drone images (Online Resource A, Fig. A.1). As agroforests with dense shade-tree canopies can hardly be differentiated from secondary forests in the satellite images, cropland cover in this study refers exclusively to monocrops, including both tree crops (e.g., orchards and cacao monocultures) and non-tree crops (e.g., rice and corn fields). Therefore, we did not distinguish between different types of monocrops in the calculation of cropland cover, which limits our conclusions about how bat assemblages were affected by the cover of specific crop systems in the surroundings of the agroforest. Non-cropland areas in these surrounding landscapes were mainly composed of secondary forests, grasslands and waterbodies. To calculate the edge density of forested/densely vegetated areas around the plots, we selected vegetation patches with > 85% tree cover and > 0.5 ha size to avoid overestimating edge density by including scattered vegetation. We calculated edge density as the perimeter of the selected patches (m) divided by the buffer area (ha) using the R package *landscapemetrics* (Hesselbarth et al. 2025). Integrating these three landscape structure variables allowed us to assess the influence of landscape composition (represented by tree and cropland cover) and configuration (represented by edge density) on the multidimensional diversity of different bat guilds (Cisneros et al. 2015).

### Estimating future landscape structure

We designed two possible future landscape scenarios for the year 2050 to assess how land-use change may influence bat diversity under either ongoing deforestation or cropland reforestation in the surroundings of the agroforests. To do so, we extracted tree cover data for the year 2000 and calculated the mean annual rate of tree cover loss from 2020 to 2024 at both spatial scales using annually updated tree cover data from Hansen et al. (2013). The ‘Deforestation scenario’ forecasts a business-as-usual situation in which the annual rate of tree cover loss around each plot will remain similar to the rate observed in the last four years. This scenario is based on recent tree cover loss data to better represent the current deforestation under international policies such as the EU Regulation on Deforestation-free Products (Regulation EU 2023/1115). Given that the European Union is one of the largest buyers of Peruvian agricultural products (e.g., 35% Peru’s cacao exports in the first quarter of 2025 went to the European Union; MINCETUR 2025), this new regulation could have a significant impact on the country´s agricultural landscapes. Tree cover loss in the deforestation scenario can be caused by cropland expansion into tree-covered areas (with an increase in cropland cover equivalent to the loss of tree cover outside existing cropland areas) and the intensification of the current croplands (tree cover loss inside existing cropland areas; Online Resource A, Fig. A.2).

Conversely, the ‘Reforestation scenario’ predicted the recovery of the tree cover lost from 2000 to 2020, either by transforming monocrops into agroforestry systems or by active reforestation of cropland areas, thus reducing the existing cropland cover in a similar percentage than the increase in tree cover (Online Resource A, Fig. A.2 & Table A.1). Therefore, we do not consider the reforestation of non-cropland areas in this scenario to keep the balance between the increase in landscape tree cover and the decrease in landscape cropland cover in the estimation of future landscape composition. The reforestation scenario represents a future in which policymakers incentivize diversified farming systems such as agroforests and tree recovery initiatives aligned with the aims of the UN Decade on Ecosystem Restoration (https://www.decadeonrestoration.org/). For both scenarios, we calculated the expected tree and cropland cover in 250 and 500 m buffers around each plot for the year 2050 and then estimated the future edge density in plot surroundings based on the future tree cover using Generalized Additive Models (see Online Resource A for details).

### Bat sampling

We sampled aerial insectivores using Song Meter Mini Bat recorders (Wildlife Acoustics Inc., USA). As year-round sampling was not feasible due to logistical constraints, we sampled aerial insectivores in the transition between the dry and wet seasons (August-November 2022) to record species that may vary in activity between seasons (Ferreira et al. 2017). This allowed us to maximize acoustic detectability of the species and obtain a more complete representation of their assemblages. Each of the 28 plots was sampled from sunset (18:00) to midnight (00:00) for four nights. These were divided into two sampling sessions of two consecutive nights spaced by an average of 39 days (20 days SD) between sessions (total sampling effort = 112 recording nights; 672 h). We placed the recorders at 1.5 m above ground and changed their location between sessions to partially capture the spatial heterogeneity of the plot. Bat recorders were programmed to be triggered by ultrasonic sounds and record for five seconds (minimum trigger frequency = 12 kHz; sampling rate = 385 kHz; left channel gain = 12 dB).

We used Kaleidoscope Pro v.5.6.6. (Wildlife Acoustics, USA) to identify recordings containing bat calls with 8–180 kHz frequency, 2–500 ms length and less than 500 ms between pulses. We visualized the spectrograms of all the recordings identified by the software to discard false detections and identify ‘bat passes’ (five-second recordings containing at least two echolocation calls of the same species) following Arias-Aguilar et al. (2018) and Lopez-Baucells et al. (2016). Echolocation calls that could not be identified at the species level were classified into sonotypes (groups of species with similar echolocation call shape and frequency). Additionally, we manually identified feeding buzzes (fast-pulse echolocation calls used by aerial insectivores in hunting attempts) to estimate aerial insectivore feeding activity.

Phyllostomids were sampled using mist nets because most phyllostomid species have similar echolocation calls and cannot be effectively sampled using bat recorders (Yoh et al. 2020). Mist-netting was carried out in the transition between the wet and dry seasons (March–July 2023) to capture species that may vary in abundance between seasons and obtain a more comprehensive representation of species assemblages (Ferreira et al. 2017). In this case, we only sampled 16 of the 28 plots due to logistical constraints imposed by the greater effort required for this methodology. The 16 plots (eight per region) were selected to represent a land-use intensity gradient within each region. We sampled each plot from sunset (18:00) to midnight (00:00) for four non-consecutive nights using four ground mist nets (12 × 2.5 m; 20 mm mesh size; total sampling effort = 64 mist-netting nights; 7680 mist-net hours x m^2^). Visits to the same plot were spaced in time by at least two weeks. The four nets were deployed in two pairs of two consecutive nets with a 90° angle (L-shape) along corridors between two rows of cacao trees. Both pairs of nets were located close to the center of the plot and separated by approximately 25 m. Mist nets were checked every 20 min. Each bat captured was measured, identified following Díaz et al. (2021) and Lopez-Baucells et al. (2016), and released after marking them through hair clipping. Bats were processed in compliance with the guidelines of the American Society of Mammalogists (Sikes et al. 2016). Aerial insectivores captured in the mist nets were excluded from the analyses, as they cannot be properly sampled with this methodology (Carvalho et al. 2023).

### Bat diversity metrics

We quantified aerial insectivore and frugivore diversity at the taxonomic, functional and phylogenetic level (Table 1). For this, we classified phyllostomids as frugivorous if at least 70% of their diet was composed of fruits (Wilman et al. 2014). Taxonomic diversity was calculated as the species richness recorded or captured per night and the Simpson diversity (Hill number q = 2) to represent the diversity of the assemblage giving more weight to dominant species. We also calculated frugivore abundance (number of individuals captured, excluding same-night recaptures) and aerial insectivore activity (number of bat passes recorded) per night. Aerial insectivorous species with less than four passes in the night were not considered when calculating aerial insectivore taxonomic, functional or phylogenetic diversity to avoid overrepresenting species that might have been recorded while commuting through the plot or adjacent areas (Online Resource B, Table B.1).

**Table 1:** Summary of response variables used to assess landscape effects on aerial insectivorous and phyllostomid bat assemblages in cacao agroforests

Response variable	Bat group	Related diversity dimension/ecosystem function
No. species recorded/captured (species richness)	Aerial insectivores & frugivores	Taxonomic diversity
Simpson diversity (q = 2)	Aerial insectivores & frugivores	Taxonomic diversity
No. bat passes	Aerial insectivores	Activity
No. frugivorous bats captured	Frugivores	Abundance
No. feeding buzzes (all spp.)	Aerial insectivores	Potential pest control
No. feeding buzzes (understory spp.)	Aerial insectivores	Potential pest control
No. feeding buzzes (canopy spp.)	Aerial insectivores	Potential pest control
No. small frugivorous bats captured	Frugivores	Potential seed dispersal
No. large frugivorous bats captured	Frugivores	Potential seed dispersal
No. insect-eating bats captured	Non-frugivorous phyllostomids	Potential pest control
Standardized effect size of the functional richness (SES.FRic)	Aerial insectivores & frugivores	Functional diversity
Standardized effect size of the mean pairwise distance (SES.MPD)	Aerial insectivores & frugivores	Phylogenetic diversity

Aerial insectivores were classified into understory or canopy-foraging species based on literature (see Table 2 for references). Small frugivores weighed < 30 g; large frugivores ≥ 30 g (mean values per species). Insect-eating phyllostomids included species with ≥ 30% of their diet composed of invertebrates

We calculated additional response variables as indicators of ecosystem functions of each bat group. For aerial insectivores, we calculated the number of feeding buzzes recorded per night to analyze landscape effects on their potential pest control services. Feeding buzzes were calculated for the complete aerial insectivore assemblage, and canopy and understory foraging species separately. Aerial insectivore species were classified into canopy or understory foragers based on existing literature (Table 2). For frugivores, we calculated the abundance of small (< 30 g) and large (≥ 30 g) species to estimate landscape influence on the dispersal of seeds of different sizes based on the positive correlation between body mass of the disperser and the size of the seeds it disperses (Sivault et al. 2023). Additionally, we used the abundance of insect-eating phyllostomids (including insectivorous, omnivorous and nectarivorous phyllostomids with at least 30% of the diet composed of invertebrates; Wilman et al. 2014) as an indicator of phyllostomid-mediated arthropod suppression (Table 2). By gleaning arthropods from leaves and branches, insect-eating phyllostomids could potentially consume phytophagous arthropods damaging cacao trees and provide important pest control services in agroforests and other agricultural systems (Kunz et al. 2011). We did not analyze landscape effects on nectarivorous species separately due to the low number of captures, which were highly concentrated in plots that might have been close to roosting sites.

**Table 2:** List of functional traits of aerial insectivores and frugivores used to estimate bat functional diversity in cacao agroforests

Functional trait	Bat group	Data type	Source
Forearm length (mm)*	Both	Continuous	Phyllostomids: our data Aerial insectivores: (Conenna et al. 2021)
Body mass (g)*	Both	Continuous	Phyllostomids: our data Aerial insectivores: Conenna et al. (2021)
Wing loading *	Both	Continuous	Conenna et al. (2021)
Wing aspect ratio	Both	Continuous	Conenna et al. (2021)
Vertical strata	Both	Categorical (understory or canopy)	Conenna et al. (2021); Marques et al. (2016); Mas (2014); Rex et al. (2011)
Frequency of maximum energy (kHz)	Aerial insectivores	Continuous	Our data
Call shape	Aerial insectivores	Categorical (CF, CF-FM, CF-FM-CF, FM-QCF or QCF)	Arias-Aguilar et al. (2018); Lopez-Baucells et al. (2016)
% of invertebrates in diet **	Phyllostomids	Continuous (%)	Wilman et al. (2014)
% of fruit in diet **	Phyllostomids	Continuous (%)	Wilman et al. (2014)
% of nectar in diet **	Phyllostomids	Continuous (%)	Wilman et al. (2014)

Functional trait values can be found in Tables B.1 and B.2 in the Online Resource B. Traits marked with * were correlated and grouped to calculate Gower´s distance between species. Traits marked with ** were grouped and treated as fuzzy traits to calculate Gower´s distance. Call shapes: *CF* constant frequency, *QCF* quasi-constant frequency, *FM* frequency modulated. Calls may be formed of different types of frequencies (e.g., a CF-FM call starts with a constant frequency and ends with a frequency modulated)

We selected functional traits reflecting key ecological functions and foraging behaviors to characterize the functional diversity of aerial insectivores and frugivores using both information extracted from mist-netting and literature (Table 2). We used five functional traits for both bat groups, which capture general characteristics of their body and foraging preferences, namely: forearm length, body mass, wing loading and aspect ratio, and preferred vertical strata (Table 2; Online resource B). Bat species with small body sizes and/or low wing loading and aspect ratios, as well as those foraging in the understory, have limited dispersal ability and may be more negatively affected by cropland expansion and tree cover loss (Colombo et al. 2023; Farneda et al. 2015; García-Morales et al. 2013; Núñez et al. 2019). Given the large differences in ecological requirements and functions of aerial insectivores and frugivores, other traits were exclusive to each group. For aerial insectivores, we included two additional echolocation traits (frequency of maximum energy, also known as peak frequency, and call shape, i.e., the shape of the echolocation call in the spectrogram) related to space use and foraging mode (Table 2). High-frequency echolocation calls and those with specific shapes (e.g., constant frequency calls; Online Resource B) are typical of species foraging in cluttered environments, which may decrease in abundance with tree cover loss and monoculture expansion (Jones 1999; Jones & Holderied 2007). For frugivores, we used three additional traits related to dietary composition as a proxy of dietary specialization, namely: percentages of invertebrates, fruit and nectar (Table 2). Frugivorous species that exploit a wide variety of food resources are expected benefit from the increased resource availability typically found in fragmented landscapes with high tree cover and edge density (Bonaccorso 1979). A more detailed description of the above-mentioned functional traits and their calculation can be found in the Online resource B.

We calculated the functional distance between species for each bat group separately using a corrected version of Gower´s distance that equalizes the contribution of correlated traits (Table 2) with the *gawdis* R package (Bello et al. 2021). The resulting distance matrices were used to estimate the functional richness (FRic) as the functional convex hull volume of the species assemblages captured/recorded in each plot and night with the *mFD* package (Magneville et al. 2022). We used null models to calculate the standardized effect size of the FRic (SES.FRic) and remove the influence of species richness on FRic (Mason et al. 2013). Negative SES.FRic values indicate lower functional richness than expected by chance, an indication of environmental filtering. On the other hand, positive SES.FRic values indicate greater trait differences between species than expected by chance, indicating niche partitioning (Swenson 2014). Positive SES.FRic values are expected in heterogeneous landscapes with high resource diversity. Conversely, in more continuous landscapes, we would expect to find assemblages of species with traits adapted to the dominant land-use, resulting in negative SES.FRic values.

We characterized the phylogenetic structure of aerial insectivore and phyllostomid assemblages using a resolved majority-rule consensus phylogenetic tree for the detected species based on 1000 trees extracted from of Upham et al. (2019; Online Resource B, Fig. B.1). As with functional diversity analyses, the most probable species of each unidentified sonotype was included in the tree (Online Resource B, Table B.1). We calculated the mean pairwise phylogenetic distance (MPD) between the species assemblages recorded in each plot and night to estimate their phylogenetic diversity using the R package *picante* (Kembel et al. 2020). We then removed the influence of species richness on MPD values by calculating the standardized effect size of the MPD (SES.MPD; Swenson 2014). Positive SES.MPD values indicate bat assemblages are composed of more distantly related species than expected by chance (phylogenetic overdispersion), while negative values indicate more closely related species (phylogenetic clustering). Negative SES.MPD are expected in intensively managed landscapes due to the selection of closely-related, disturbance-adapted species (hypothesis 3).

SES.MPD and SES.FRic values below -1.96 or above 1.96 indicate significant phylogenetic and functional clustering or overdispersion, respectively (Swenson 2014). Both SES.FRic and SES.MPD are suitable indices for assessing land-use effects on the functional and phylogenetic structure of ecological communities because of their independence of species richness and their ability to detect filtering/overdispersion patterns (Mason et al. 2013; Tucker et al. 2017). See Online Resource B for details on the calculation of SES.FRic and SES.MPD.

### Statistical analyses

We used mixed models (LMMs and GLMMs) to analyze the effect of tree cover, cropland cover and edge density in the surroundings of each plot (henceforth called landscape predictors) on the different metrics of bat diversity in each region (Table 1). Before modeling, we assessed plot-level sample coverage using the R package iNEXT (Hsieh et al. 2022) and scaled and centered landscape predictors (mean = 0; SD = 1). Models were fitted using the *glmmTMB* package (Brooks et al. 2022) in R v.4.1.2 (R Core Team 2025). We used Gaussian distributions (for continuous response variables: Simpson diversity, SES.FRic and SES.MPD) or Poisson distributions (count response variables: species richness, abundance, activity and number of feeding buzzes of each guild; Table 1). We included the plot identity (for phyllostomids) and the sampling session nested within the plot identity (for aerial insectivores) as random effects to account for spatiotemporal dependencies.

Species assemblages in more changing and disturbed regions are usually more adapted to landscape intensification (Hua et al. 2024). To test for possible variations of the landscape effects between the intensive and non-intensive regions, we fitted a full model containing all three landscape predictors and their two-way interactions with region identity. For each response variable, we used the Akaike Information Criterion corrected for small sample sizes (AICc) to compare models fitted with landscape predictors measured at 250 and 500 m to select the spatial scale that best fitted the data. When the two models were equal (∆AICc < 2), we selected the models fitted using 500 m buffers for a wider landscape representation. We then extracted the p-values of the landscape effect in each region from the summary of the selected model by changing the reference region in the model. We checked model assumptions (uniformity and overdispersion of the residuals, normality of the random effects, collinearity of the predictors and zero-inflation) using the R package *performance* (Lüdecke et al. 2023). In case of significant overdispersion in the Poisson models, we used a Negative Binomial distribution. Collinearity between predictors was assessed using the Variance Inflation Factor (VIF). Finally, we tested for spatial autocorrelation of the residuals using Moran’s I test (Paradis et al. 2024).

Models showing significant landscape effects were projected to the two future landscape scenarios mentioned in Sect. “Estimating future landscape structure”. To better reflect the observed variability in land-use trends within each region, model projections were performed at the plot level, with future tree and cropland cover and edge density calculated for each plot separately. If the landscape effects were significant only in one region, we report model projections for that specific region only. Finally, we compared the predicted values of the response variables for the current and future landscape scenarios using paired Wilcoxon signed rank tests to evaluate future risks and conservation opportunities for bat diversity and services. It is important to note that our model projections are based on the registered bat assemblages under the current landscape structures. Therefore, our forecasts may be less reliable for plots predicted to fall outside the observed ranges of landscape predictors in the future (Table A.1). Given the non-linear trajectories of land-use change, these analyses are intended as a scenario exploration to highlight potential sensitivities in certain species groups rather than precise forecasts of future bat assemblages.

## Results

### Aerial insectivore and phyllostomid assemblages

We registered at least 55 bat species in total, 26 aerial insectivore species/sonotypes and 29 phyllostomids species. For the aerial insectivores, we recorded 54,111 bat passes from 19 species and 7 unidentified sonotypes (hereafter just species, for simplicity), belonging to six families (see Table C.1 in Online Resource C for a list of species). Two species were recorded only in the intensive region (33,300 passes in total) and one only in the non-intensive one (20,811 passes in total). *Myotis nigricans* was the most recorded aerial insectivore in both regions, accounting for 59.4% and 25.6% of the passes recorded in the intensive and non-intensive regions, respectively. Aerial insectivore sample coverage was above 99% in all plots (Fig. C.1 in Online resource C).

We captured 1,645 phyllostomids belonging to 29 species, excluding same-night recaptures. Of these, 16 species were classified as frugivores (1,335 individuals) and 12 species as insect-eating phyllostomids (306 individuals). The remaining species was *Desmodus rotundus*, a sanguivore phyllostomid (four individuals captured). Three phyllostomid species were captured only in the intensive region (658 captures in total), and 10 only in the non-intensive one (987 captures in total; see Table C.2 in Online Resource C for a list of species). The frugivorous species *Artibeus lituratus* and *Carollia perspicillata* were the most common phyllostomids in the intensive and non-intensive regions, respectively. Together, they accounted for 50.8% of the total number of captures (62.5% of the frugivores captured). Phyllostomid sample coverage was above 95% in all plots but one in the intensive region with 87.1% and one in the non-intensive with 92.6% (Fig. C.1 in Online resource C).

### Landscape effects on aerial insectivores

**Fig. 2:**
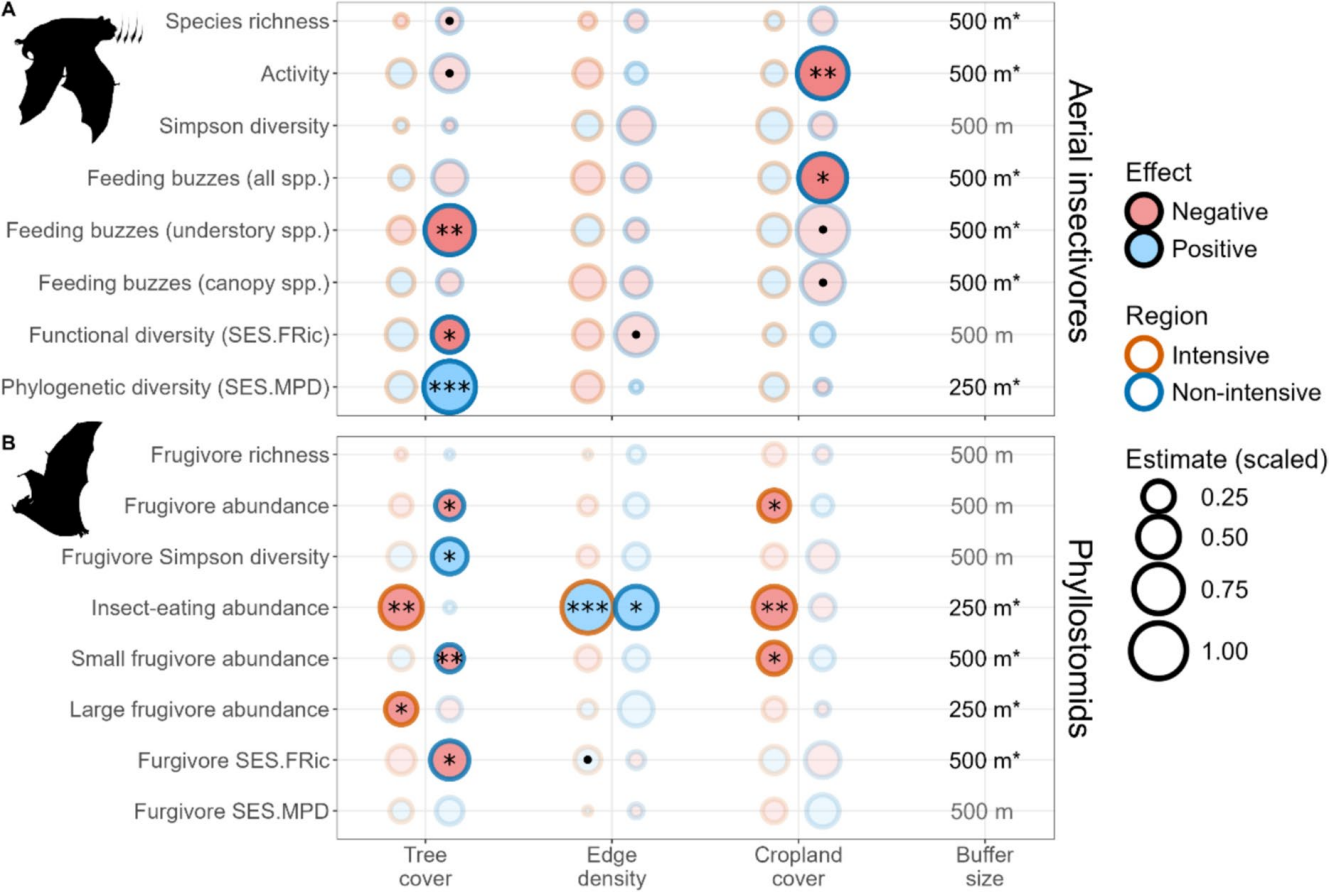
Summary of landscape effects on aerial insectivores and phyllostomids and selected buffer sizes. Positive effects are represented by blue circles, negative effects by red circles. Transparent circles represent non-significant or marginally significant effects. Symbols inside the circles indicate the level of significance (•*p* < 0.1; *p < 0.05, **p < 0.01, ***p < 0.001). The outline of the circles indicates the regional context (intensive region: orange outline; non-intensive region: blue outline). Bubble sizes represent the size of the effect based on the scaled model estimates (specific *p*-values and estimates can be found in Table D.3 in Online Resource D). Note that scaled estimates are only comparable within response variables. Buffer sizes marked with asterisks outperformed the alternative size (∆AICc > 2)

In case of aerial insectivores, models with landscape predictors computed in 500 m buffers around the plot outperformed those with smaller buffers of 250 m (∆AICc > 2) in five of the eight response variables (species richness, activity, and number of feeding buzzes of the complete assemblage, and understory and canopy species separately; Fig. 2; Table D.1 in Online Resource D). Aerial insectivore phylogenetic diversity (SES.MPD) was the only variable better explained by landscape predictors within 250 m buffers, while there was no difference (∆AICc < 2) between 500 and 250 m models for Simpson diversity and functional diversity (SES.FRic).

**Fig. 3:**
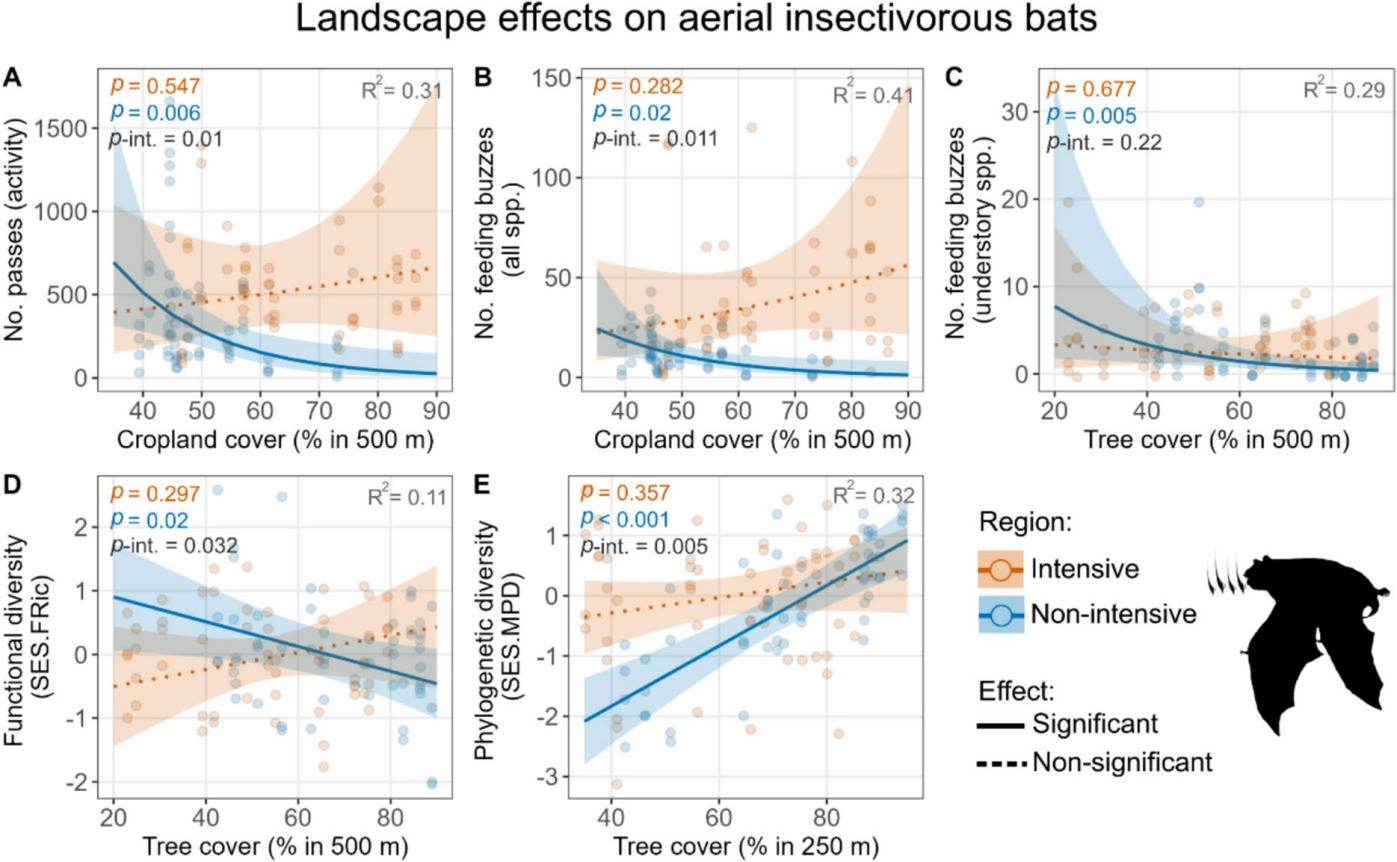
Effects (trends and 95% CI) of landscape predictors on the activity (**A**), feeding buzzes (**B**, **C**), functional (**D**) and phylogenetic (**E**) diversity of aerial insectivorous bats. Orange lines and CIs represent trends in the intensive region, blue lines and CIs trends in the non-intensive one. Solid lines indicate significant trends (*p* < 0.05). Points represent the raw values. For better visualization, we removed two points above 2000 in **A**, five points above 150 in **B** and one point above 30 in **C** and **D**. Non-significant relationships not included here can be found in Figure D.1 in Online Resource D

Five of the eight response variables of aerial insectivores were significantly affected by landscape predictors only in the non-intensive region, while none were affected in the intensive region (Fig. 2; Table D.2 in Online Resource D). Aerial insectivore activity decreased with cropland cover in the non-intensive region, dropping from an average of about 500 passes per night at 40% cropland cover to only 25 passes at 90% (Fig. 3A; Table D.3 in Online Resource D). The number of feeding buzzes also decreased with cropland cover in the non-intensive region due to marginal decreases in the feeding buzzes of both understory and canopy species (Fig. 3B, D.1 in Online Resource D). Additionally, the feeding buzzes of understory aerial insectivores decreased with increasing tree cover in the non-intensive region (Fig. 3C).

The functional diversity (SES.FRic) of aerial insectivores was negatively affected by landscape tree in the non-intensive (Fig. 3D). However, we did not find signs of functional clustering or overdispersion (-1.96 < SES.FRic < 1.96; Fig. 3D).

Conversely, the phylogenetic diversity (SES.MPD) of aerial insectivores was positively correlated with the tree cover in the 250 m buffer around the plot in the non-intensive region (Fig. 3E). Here, aerial insectivore assemblages in more open landscapes (35–40% of tree cover) were mainly composed of closely related species (phylogenetic clustering; SES.MPD < − 1.96). None of the aerial insectivore or phyllostomid models showed spatial autocorrelation in the residuals (Table D.4 in Online Resource D). VIF values showed low to moderate multicollinearity for landscape predictors in the aerial insectivore models (VIF < 10; Table D.5 in Online Resource D).

The random effects of plot identity and sampling round explained a high proportion of the variability in the response variables overall, with marginal R2 values ranging from 0.03 to 0.4, while conditional R2 values ranged from 0.42 to 0.9 (Table D.1 in Online Resource D).

### Landscape effects on frugivores and insect-eating phyllostomids

The abundance of insect-eating phyllostomids and large frugivores were better modeled with landscape predictors computed for 250 m buffers than 500 m buffers (Table D.1 in Online Resource D). Conversely, frugivore functional diversity and the abundance of small frugivores were better modeled when using landscape predictors measured in 500 m buffers. We did not find differences between the two spatial scales for the overall frugivore richness, abundance, Simpson diversity and phylogenetic diversity (∆AICc < 2), and therefore, used 500 m buffers to model these response variables.

**Fig. 4:**
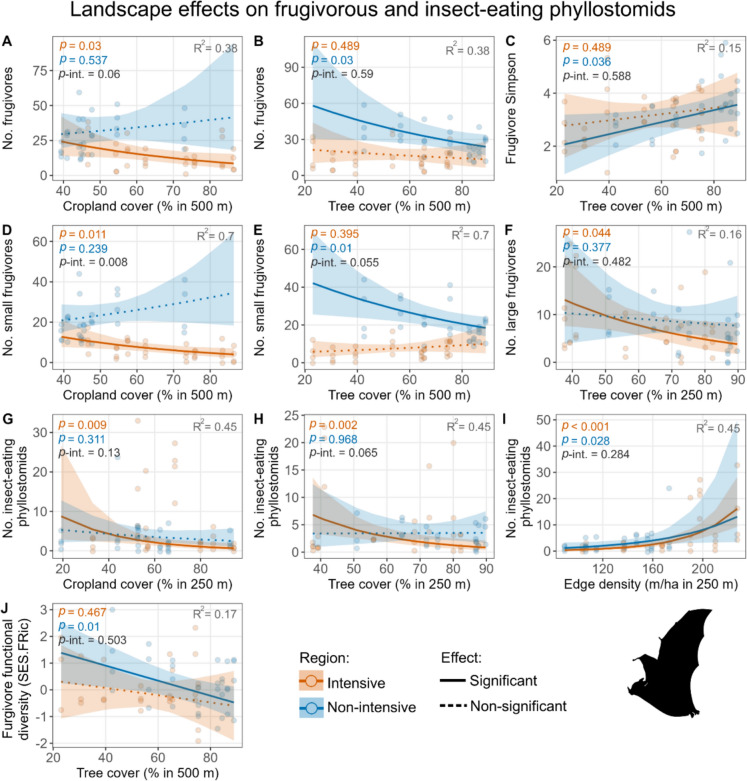
Effects (trends and 95% CI) of landscape predictors on the abundance (**A**, **B**) and Simpson diversity (**C**) of the complete frugivore assemblage, the abundance of different functional guilds (**D**–**I**) and frugivore functional diversity (SES.FRic). Orange lines and CIs represent trends in the intensive region, blue lines and CIs trends in the non-intensive region. Solid lines indicate significant trends (*p* < 0.05). Points represent the raw values. Non-significant relationships not included here can be found in Figure D.2 in Online Resource D

Frugivore abundance decreased with increasing cropland cover in the intensive region due to the decrease in the abundance of small-sized species (Figs. 4A, D). Additionally, frugivore abundance in agroforests of the non-intensive region decreased from ca. 60 to 24 captures with increasing tree cover in the surrounding landscape, also due to lower abundances of small-sized frugivores (Figs. 4B, E). Conversely, frugivore Simpson diversity increased with increasing tree cover in 500 m buffers in the non-intensive region (Fig. 4C), while the abundance of large-sized frugivores decreased with increasing tree cover in the intensive region (Fig. 4F). Finally, insect-eating phyllostomid abundance decreased with increasing cropland and tree cover in the intensive region and increased with higher edge density in both regions (Figs. 3G–I).

Frugivore functional diversity (SES.FRic) was negatively associated with higher landscape tree cover in the non-intensive region, although predicted values stayed within the − 1.96 to 1.96 range, indicating a lack of significant functional clustering or overdispersion (Fig. 4J).

Overall, the random effect of plot identity explained a low proportion of the variability in the frugivore and insect-eating phyllostomid response variables, which was mainly explained by the fixed effects (marginal R2: 0.06–0.7; conditional R2: 0.06–0.7; Table D.1 in Online Resource D).

### Aerial insectivores under future scenarios

**Fig. 5:**
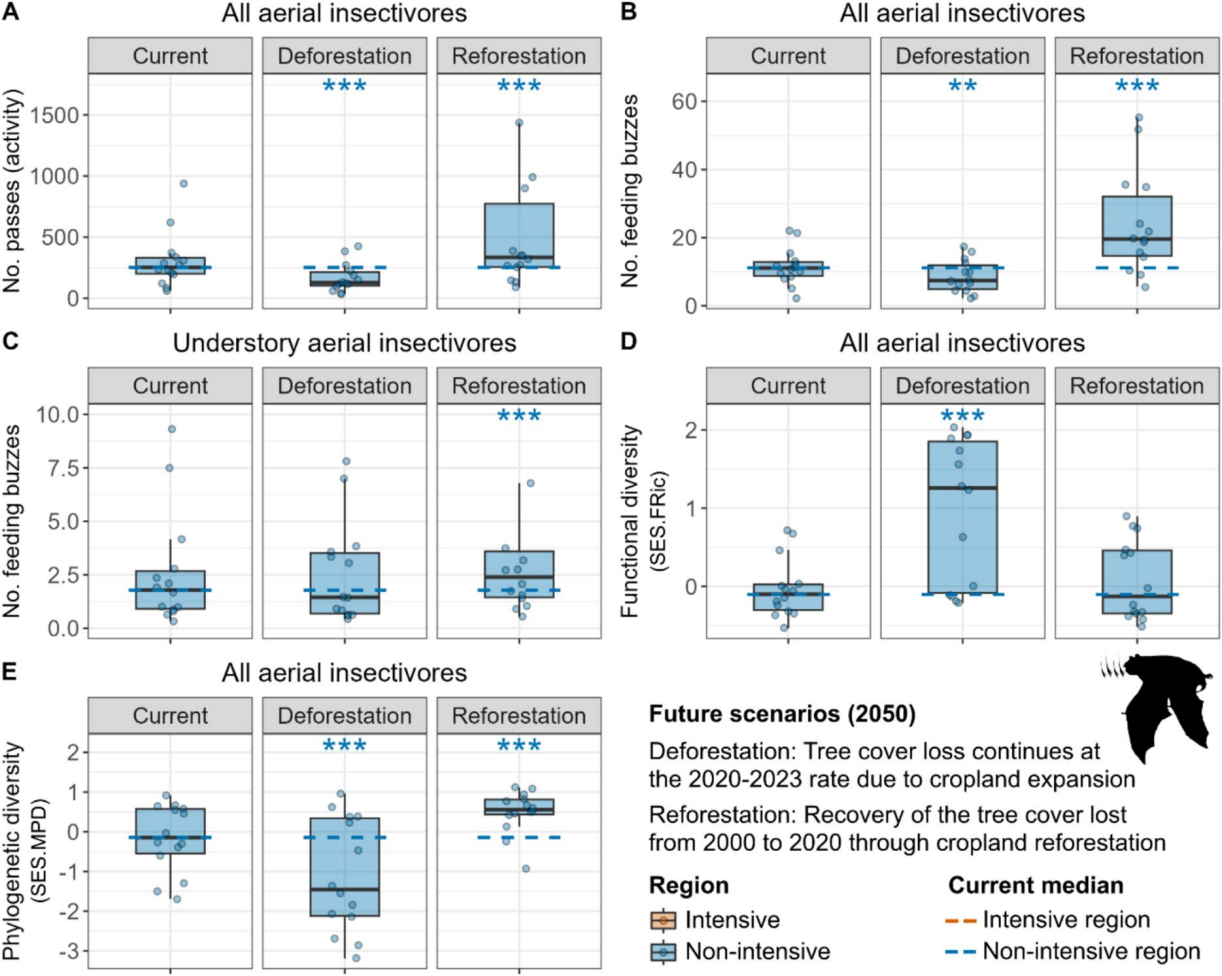
Boxplots showing the distribution of predicted values of aerial insectivore activity (**A**), feeding buzzes (**B**–**C**), functional (**D**), and phylogenetic (**E**) diversity per plot (points) for the current and future landscape scenarios. Asterisks indicate significant deviations from the current scenario (**p < 0.01, ***p < 0.001; Wilcoxon signed rank test results can be found in Table D.6 in Online Resource D). Dashed lines mark the median of model predictions under the current landscape scenario. One point above 1,500 in A (reforestation scenario) and three points above 10 in C (reforestation scenario) were removed from the graphs for better comparison of the boxplots (boxplot shapes were not modified)

We projected five aerial insectivore response variables significantly influenced by landscape variables on future landscape scenarios: activity, feeding buzzes of the complete assemblage and understory species only, functional and phylogenetic diversity (Fig. 5). Model projections of aerial insectivores were limited to plots in the non-intensive region, as none of the response variables was affected by landscape predictors in the intensive one (Fig. 2). Under maintained deforestation, aerial insectivore activity and the number of feeding buzzes of the complete assemblage and understory species are expected to decrease significantly (Fig. 5A–C). Deforestation is also expected to increase the functional diversity and reduce the phylogenetic diversity of aerial insectivore assemblages in the non-intensive one (Fig. 5D, E). Model projections predicted higher activity and more feeding buzzes of the overall assemblage and understory species with cropland reforestation in the non-intensive region. Additionally, reforestation would increase aerial insectivore phylogenetic diversity in this region.

### Frugivores and insect-eating phyllostomids under future scenarios

**Fig. 6:**
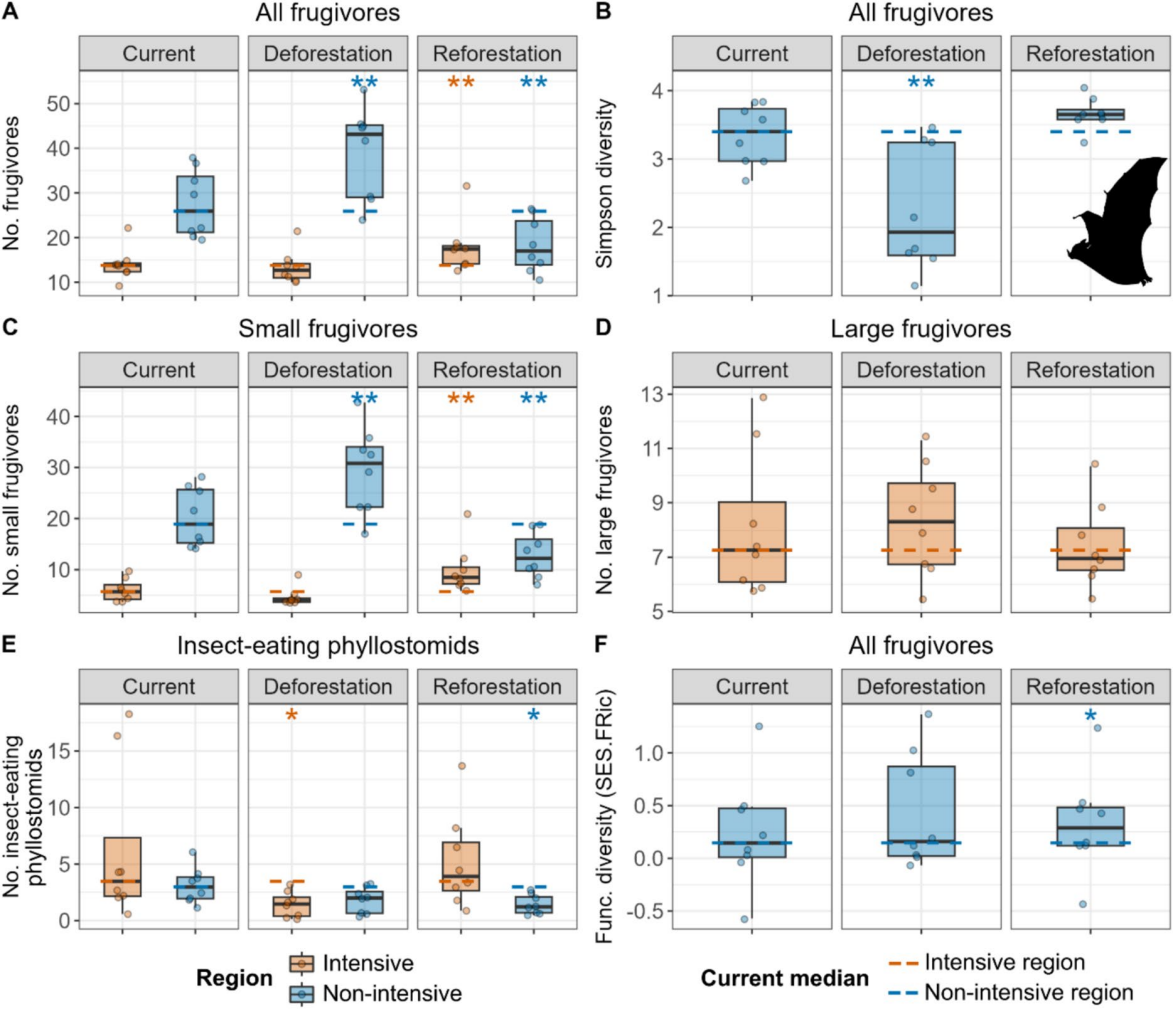
Boxplots showing the distribution of the predicted values of abundance (**A**) and Simpson diversity (**B**) of the complete frugivore assemblage, abundance of small (**C**) and large (**D**) frugivores, abundance of insect-eating phyllostomids (**E**) and frugivore functional diversity (**F**) per plot (points) for the current and future landscape scenarios. Asterisks indicate significant deviations from the current scenario (*p < 0.05, **p < 0.01; Wilcoxon signed rank test results can be found in Table D.6 in Online Resource D), and colors distinguish the intensive (orange) and non-intensive regions (blue). Dashed lines mark the median of model predictions under the current landscape scenario

Phyllostomid model projections were limited to six response variables significantly affected by landscape predictors: Abundance, Simpson diversity and functional diversity of the complete frugivore assemblage, the abundance of small and large frugivores and the abundance of insect-eating phyllostomids (Fig. 2). Deforestation was predicted to increase the abundance of the overall frugivore assemblages in the non-intensive regiondue to higher abundances of small-sized species (Fig. 6A, C), but decrease the abundance of insect eating phyllostomids in the intensive region (Fig. 6E). Additionally, deforestation would also reduce the Simpson diversity of the frugivore assemblage in both regions (Fig. 6B). Cropland reforestation is expected to increase the overall frugivore abundance in the intensive region by increasing the abundance of small frugivores, but reduce the abundance of the overall frugivore assemblage, small frugivores and insect-eating phyllostomids in the non-intensive region. Additionally, cropland reforestation would lead to higher functional diversity of the frugivore assemblage in the non-intensive region (Fig. 6F), while the abundance of large furgivores is not expected to vary under the future deforestation or reforestation scenarios (Fig. 6D).

## Discussion

We assessed how aerial insectivorous and frugivorous bats in Amazonian cacao agroforests responded, at the taxonomic, functional and phylogenetic levels, to land-use intensification in the landscape surrounding the agroforest. In line with our first hypothesis, aerial insectivore activity and the number of feeding buzzes decreased with higher cropland cover in the non-intensive region, but not in the intensive one. Conversely, increasing cropland cover resulted in lower abundances of frugivorous and insect-eating phyllostomids in the intensive region only. Contrary to our second hypothesis, which predicted higher frugivore and insectivore abundance with increasing landscape tree cover, we found negative effects of landscape tree cover on frugivore abundance and positive effects of edge density on insect-eating phyllostomids in both regions. Moreover, the number of feeding buzzes of understory aerial insectivores decreased with increasing tree cover in the non-intensive region. Finally, in line with our third hypothesis, decreasing landscape tree cover resulted in higher phylogenetic homogenization of aerial insectivore assemblages and lower frugivore Simpson diversity in the non-intensive region. Our results indicate that preserving bat diversity and aerial insectivore ecosystem functions in Amazonian agricultural areas relies on retaining and restoring tree cover at the landscape level.

### Agricultural intensification effects on aerial insectivores

Aerial insectivore assemblages in cacao agroforests were negatively affected by cropland expansion and tree cover loss in the non-intensive region, but not in the intensive one, possibly due to stronger ecological filtering by past deforestation and higher seasonality in the latter (Hua et al. 2024). Species assemblages in seasonally dry forests and regions subjected to longer deforestation history are usually more adapted to environmental disturbances and have high dispersal ability and changing diets, allowing them to exploit ephemeral resources (Hua et al. 2024; Stoner & Timm 2011). This is reflected by the dominance of disturbance-tolerant species like *Myotis nigricans* in the intensive region (60% of passes), while dense-vegetation foraging *Pteronotus* species dominated in the non-intensive region (41% of passes). Our results also indicate that cropland expansion in the non-intensive region could reduce aerial insectivore feeding buzzes in cacao agroforests. Thus, prioritizing conservation interventions limiting monoculture expansion may be essential to preserve aerial insectivorous bats and their ecosystem functions in low-intensity agricultural landscapes. Disentangling how land-use intensity, seasonality and agricultural history influence bat assemblages in Neotropical agricultural areas could help refine tailored management strategies aimed at promoting their conservation and ecosystem functions in these landscapes.

Both understory- and canopy-associated aerial insectivores were partially affected by cropland cover, probably due to pesticide and herbicide applications in the surrounding monocrops that reduced arthropod abundance. Contrary to our expectations, the feeding buzzes of understory aerial insectivores decreased with increasing landscape tree cover in the non-intensive region. Here, non-crop habitats with low tree cover were mainly cattle pastures and other open areas such as waterbodies and sandy riverbanks. The lack of understory vegetation in these land types might concentrate understory aerial insectivore foraging efforts in cacao agroforests, as happens with phyllostomids in other Neotropical cacao-growing landscapes (Ocampo-Ariza et al. 2022). These results further highlight the importance of agroforestry systems for biodiversity conservation in tropical agricultural areas (Maas et al. 2020; Tscharntke et al. 2011).

As in previous studies in the Brazilian Atlantic forest, landscape intensification did not significantly affect aerial insectivore species richness (Falcão et al. 2021), but it influenced their functional and phylogenetic diversities. Aerial insectivore functional diversity decreased with increasing tree cover in the non-intensive region. Densely forested matrices may prevent species with limited ability to fly in cluttered environments from reaching the agroforests, filtering out species with high wing loading and aspect ratio and low-frequency echolocation calls and reducing the functional diversity of the local assemblage (Cisneros et al. 2015; Norberg et al. 1997). This result might have also been influenced by reduced detectability of species flying at higher heights in more forested areas, highlighting a potential limitation of bat assessments using passive acoustic monitoring techniques (Roemer et al. 2025). However, functional diversity values were within the expected range, indicating that landscape effects were not strong enough to cause trait-based environmental filtering of aerial insectivore assemblages (Swenson 2014).

Conversely, tree cover loss in the surroundings of the agroforest resulted in the phylogenetic clustering of aerial insectivore assemblages in the non-intensive region. These assemblages were mainly composed of closely related species, possibly belonging to the Molossidae and Vespertilionidae families, which are generally little affected by habitat disturbances (Bader et al. 2015; Estrada-Villegas et al. 2010). In line with our findings, the adaptation of aerial insectivores to human-modified habitats was found to be phylogenetically conserved in African bats, with most Molossid and Vespertilionid species either exploiting anthropogenic habitats or being adapted to them (Marsden et al. 2023). The high variety of sizes, wing morphologies and echolocation call frequencies within the Molossidae and Vespertilionidae families may further explain the higher functional diversity found in agroforests in more open landscapes. Increasing land-use intensity has been reported to cause phylogenetic erosion of bat assemblages in other human-modified Neotropical landscapes (Aninta et al. 2019; Farneda et al. 2024). Our results further highlight the importance of preserving forest fragments and restoring tree cover in agricultural landscapes to enhance the conservation of bat evolutionary lineages in the Amazon region.

### Agricultural intensification effects on frugivores and insect-eating phyllostomids

Our findings indicate that, although frugivorous phyllostomids can thrive in disturbed areas, their diversity and abundance in cacao agroforests may be threatened by overly intensive land-use systems in the surrounding landscape (Willig et al. 2007). Contrary to our first hypothesis, frugivore assemblages were negatively affected by cropland cover in the intensive region, but not in the non-intensive one. While most of the cropland cover in the non-intensive region was composed of banana and cacao plantations; in the intensive region, industrial rice and papaya monocultures, frequently owned by private companies, represent a great proportion of the croplands. These intensively managed monocultures lack non-crop trees and natural vegetation, providing almost no refuges or resources for frugivorous bats. The reduced resource availability and high predation risk in open monocultures might prevent small frugivores from reaching plots surrounded by intensive croplands, explaining their lower abundance in these agroforests (García-Morales et al. 2013).

Frugivore abundance increased towards disturbed landscapes with low tree cover in the non-intensive region. Here, lower frugivore Simpson diversity and higher abundance of small frugivores in agroforests embedded in cleared landscapes indicate that mainly dominant disturbance-tolerant species, such as *Carollia perspicillata*, increased in abundance with tree cover loss. Conversely, in the intensive region, it was mainly large-sized frugivores which decreased in abundance towards forested areas. In our study area, secondary forests were mainly found in landscapes with high tree cover, while scattered trees and productive systems such as agroforests and orchards represented most of the tree cover in the most cleared landscapes. These changes in landscape tree cover composition could explain the decreased abundance of frugivores with increasing landscape tree cover. Forested matrices may be more resource-diverse and/or abundant than agroforests, causing the dilution of frugivores across the landscape. Specifically, large canopy frugivores such as those of the genus *Artibeus*, which primarily feed on figs (*Ficus* spp.; Bonaccorso 1979), may find more resources in secondary forests than in the agroforests, resulting in lower local abundances in agroforests embedded in forested matrices. On the other hand, these species may concentrate in agroforests in more open landscapes, as they provide food and refuge that are scarce in deforested matrices (Ocampo-Ariza et al. 2022; Tscharntke et al. 2012). The local concentration of species with different feeding habits and wing morphologies may have driven the observed increase in frugivore functional diversity with tree cover loss in the non-intensive region (Cisneros et al. 2015). These findings indicate that cacao agroforests can support abundant and functionally diverse assemblages of frugivorous bats in cleared and fragmented agricultural landscapes, potentially contributing to their natural regeneration through seed dispersal (Castaño et al. 2020).

The increased frugivore abundance is usually coupled with a lower abundance of insectivorous phyllostomids in more cleared habitats (Farneda et al. 2020; Medellín et al. 2000). Yet, insect-eating phyllostomid was negatively correlated with landscape tree cover in the intensive region and positively to edge density in both regions. Most insect-eating phyllostomids captured (81%) were either *Phyllostomus discolor* or *P. hastatus*, two disturbance-tolerant omnivorous species that may benefit from the higher habitat and resource diversity found in these fragmented landscapes (Cleary et al. 2016; Wilson et al. 1996). Similar positive effects of landscape-level edge density on insect-eating phyllostomids have been previously reported for other Amazonian and Central American human-modified landscapes (Chambers et al. 2016; Klingbeil & Willig 2009). However, the low abundance of purely insectivorous and carnivorous phyllostomids indicates that cacao agroforests might not be a suitable habitat for these species, as they are almost exclusively restricted to less disturbed habitats (Fenton et al. 1992; Medellín et al. 2000).

### Enhancing future bat diversity

Model projections of future landscape scenarios showed that agricultural expansion and intensification may result in phylogenetic homogenization of aerial insectivore and taxonomic homogenization of frugivore assemblages, especially in low-intensity agricultural regions. Rather than species loss, higher dominance of generalist frugivores, such as those of the genus *Artibeus* and *Carollia*, and openland aerial insectivores of the families Molossidae and Vespertilionidae would likely drive this homogenization (Aycart-Lazo et al. 2025). These results are consistent with the trends predicted by Brasileiro et al. (2022) and Gonçalves et al. (2021) for the Amazon region, where climate and land-use changes are expected to cause a replacement of habitat specialists by generalist species in the future.

Our approach for the design of future landscape structure and prediction of bat assemblages in 2050 has some limitations, especially in the deforestation scenario. Although landscape variables for this scenario were derived from observed land-use trajectories in recent years, land-use change usually follows non-linear trajectories, which are mainly driven by complex immigration processes in the Peruvian Amazon (Briceño et al. 2019). Additionally, the small spatial scales considered in this study might represent localized processes facilitating/preventing the arrival of bats to the agroforests rather than landscape-level changes in bat assemblage composition (Aycart-Lazo et al. 2025). Moreover, the predicted values of the landscape variables fell outside the observed ranges for some plots, increasing the uncertainty of these predictions. Therefore, rather than precise forecasts of future bat assemblages, these predictions should be considered as an exploration of potential general trends under plausible landscape composition scenarios to identify functional groups that may be particularly sensitive to ongoing landscape intensification and deforestation. Because reforestation can be implemented through policy measures or farmer cooperation, the predictions under this scenario are more realistic. These predictions show that landscape tree cover restoration could reverse these trends and help maintain or increase insect-eating phyllostomid abundance and aerial insectivore feeding buzzes in the intensive and non-intensive regions, respectively. Therefore, conservation actions aimed at restoring tree cover in tropical agricultural landscapes will likely result in increased bat-mediated arthropod suppression in these areas.

Transforming monocultures, such as papaya or banana, into agroforests growing native fine-flavor cacao varieties can increase landscape tree cover and enhance bat diversity while maintaining farmer livelihoods in the Western Amazon (Farneda et al. 2020; Tscharntke et al. 2023). National and regional policies recognizing the environmental sustainability of diversified farming systems and encouraging their adoption can minimize the climate and environmental impacts of food production in these regions (Ravikumar et al. 2017). Landscape tree cover restoration is expected to reduce the abundance of dominant frugivorous species in agroforests of the non-intensive region. However, rather than negatively impacting bat-mediated seed dispersal, the increased frugivore diversity in restored landscapes is expected to enhance bat-fruit network heterogeneity, making them more similar to those in continuous forests (Castaño et al. 2020). Moreover, diversifying agroforests and live fences with native fruiting trees (e.g., *Ficus*, *Spondias* or *Byrsonima* species) may attract frugivorous bats and enhance seed rain in disturbed landscapes, potentially compensating for the reduced frugivore abundance (Fremout et al. 2022; Medina et al. 2007; Pozo-Rivera et al. 2024).

Agroforestry systems and live fences provide limited resources for forest-dependent insectivorous and carnivorous bats. These species rarely persist in small forest fragments (< 100 ha) and secondary forests (Farneda et al. 2015), highlighting the importance of preserving old-grown forests to sustain diverse and multifunctional bat assemblages in Neotropical human-modified landscapes (Cleary et al. 2016; Medellín et al. 2000; Williams-Guillén & Perfecto 2010). Locally promoted initiatives, such as the creation of conservation concessions by cacao farmer cooperatives, have resulted in the protection of thousands of hectares of forest in our study areas, proving to be an effective conservation strategy (SERFOR 2013). Additionally, cropland reforestation may increase landscape tree cover above current levels, potentially attracting forest-associated insectivorous bats not detected under the current landscape structures. Together, our results provide evidence on how landscape tree cover restoration can help maintain bat genetic diversity and potential ecosystem functions, in line with the 2050 goals of the Kunming-Montreal Global Biodiversity Framework (CBD 2022). This integration of land-sharing and land-sparing strategies is increasingly necessary to enhance biodiversity and ecosystem services in Amazonian agricultural areas (Grass et al. 2019; Maeda et al. 2023).

## Conclusions

Our study shows that deforestation and land-use intensification alter bat assemblages in Amazonian cacao agroforests, with guild- and region-specific effects on functional and phylogenetic diversity, and possible consequences for ecosystem service provision. Aerial insectivorous bats were more affected by cropland expansion and tree cover loss in less disturbed regions, while phyllostomid frugivores increased in more cleared landscapes, where dominant disturbance-tolerant species thrive. These shifts indicate increasing taxonomic and phylogenetic homogenization under current land-use trends. To sustain bat-mediated pest control, conservation strategies should prioritize the retention and restoration of landscape tree cover combined with regionally tailored land-use. Our results highlight concrete landscape-level interventions, such as integrating tree cover in agricultural landscapes through agroforestry and maintaining forest fragments, that can support bat conservation and key ecosystem functions in agroforests under intensifying land-use pressures. These actions are urgently needed to prevent biodiversity erosion and secure multifunctional bat communities essential for resilient tropical agroecosystems.

## Supplementary Information

Below is the link to the electronic supplementary material.

Supplementary file1 (DOCX 5186 KB)

## Data Availability

All data for this project have been uploaded to the Open Science Framework repository at: osf.io/am6cr.
