# Supplementary material for: Tree cover loss and intensified land use simplify bat assemblages in Amazonian cacao agroforestry landscapes
Source: Landsc Ecol. 2026 May 6;41(7):115. doi: 10.1007/s10980-026-02371-6 (PMC13314815; doi:10.1007/s10980-026-02371-6)
Supplement: Supplementary file 1 — Supplementary file1 (DOCX 5186 KB) [file 10980_2026_2371_MOESM1_ESM.docx]

**Supplementary materials**

**Tree cover loss and intensified land use simplify bat assemblages in Amazonian cacao agroforestry landscapes**

**Journal:** Landscape Ecology.

**Authors:** Pablo Aycart-Lazo^1,2^* (ORCID: 0000-0002-5762-1348), Luz Sánchez-Maldonado^3,4^ (ORCID: 0000-0002-7159-9167), Blanca Ivañez-Ballesteros^2^ (ORCID: 0009-0009-4871-8162), Carolina Ocampo-Ariza^5,6,7^ (ORCID: 0000-0002-4106-5586), Stefan Dullinger^1^ (ORCID: 0000-0003-3919-0887), Evert Thomas^6^ (ORCID: 0000-0002-7838-6228), Teja Tscharntke^5^ (ORCID: 0000-0002-4482-3178), Ingolf Steffan-Dewenter^2^ (ORCID: 0000-0003-1359-3944), Bea Maas^1,8^ (ORCID: 0000-0001-9461-3243).

*^1^Department of Botany and Biodiversity Research, University of Vienna, Vienna, Austria.*

*^2^Department of Animal Ecology and Tropical Biology, Biocenter, University of Würzburg, Würzburg, Germany.*

*^3^División de Ecología Vegetal, Centro de Ornitología y Biodiversidad (CORBIDI), Lima, Peru.*

*^4^Facultad de Ciencias, Universidad Nacional Agraria La Molina, Lima, Peru.*

*^5^Functional Agrobiodiversity and Agroecology, Department of Crop Sciences, University of Göttingen, Göttingen, Germany.*

*^6^Bioversity International, Lima office, Lima, Peru.*

*^7^Ecological Networks, Technical University of Darmstadt, Darmstadt, Germany.*

*^8^Department for Integrative Biology and Biodiversity Research, Institute of Zoology, BOKU University, Vienna, Austria.*

* Corresponding author: Pablo Aycart-Lazo (pablo.aycart@univie.ac.at).

Table of contents

[**Online Resource A. Study area and future landscape scenarios** 3](#_Toc222227940)

[Table A.1. Regional differences between study areas. 3](#_Toc222227941)

[A.1. Plot characteristics 3](#_Toc222227942)

[Figure A.1. Example manual classification of satellite images 4](#_Toc222227943)

[A.2. Workflow for the calculation of future Deforestation scenario 4](#_Toc222227944)

[A.3. Workflow for the calculation of future Reforestation scenario 5](#_Toc222227945)

[A.4. Estimating future edge density values based on future landscape tree cover 5](#_Toc222227946)

[Figure A.2. Workflow used to calculate future tree and cropland cover in 2050 6](#_Toc222227947)

[Table A.2. Current and future values of landscape predictors under each scenario. 7](#_Toc222227948)

[**Online Resource B. Functional and phylogenetic diversity analyses**. 8](#_Toc222227949)

[B.1. Descriptions of traits used in the calculation of functional diversity: 8](#_Toc222227950)

[B.2. Functional diversity calculation: 10](#_Toc222227951)

[Table B.1. Functional trait values of aerial insectivorous bats. 11](#_Toc222227952)

[Table B.2. Functional trait values of phyllostomids. 12](#_Toc222227953)

[B.2. Phylogenetic diversity calculation: 13](#_Toc222227954)

[Figure B.1. Consensus phylogenetic tree used for the calculation of mean pairwise distances (MPD) 14](#_Toc222227955)

[**Online Resource C. Species lists and sample coverage evaluations.** 19](#_Toc222227956)

[Table C.1. Number of passes and feeding buzzes of aerial insectivorous bats. 19](#_Toc222227957)

[Table C.2. Number of captures of phyllostomid bats (family Phyllostomidae). 20](#_Toc222227958)

[Figure C.1. Plot-level sample coverage values 21](#_Toc222227959)

[**Online Resource D. Model selection and results**. 22](#_Toc222227960)

[Table D.1. AICc-based comparison of models at 250 and 500 m. 22](#_Toc222227961)

[Table D.2. Anova tables (Type II Wald chisquare tests) . 23](#_Toc222227962)

[Table D.3. Summary of GLMMs 27](#_Toc222227966)

[Table D.4. Results of the Moran´s I test. 35](#_Toc222227967)

[Table D.5. Variance inflation factor (VIF) . 36](#_Toc222227968)

[Table D.6. Results of Wilcoxon signed rank tests comparing current and future scenarios 40](#_Toc222227972)

[Figure D.1. Non-significant effects of landscape predictors on aerial insectivores 41](#_Toc222227973)

[Figure D.2. Non-significant effects of landscape predictors on phyllostomids. 42](#_Toc222227974)

# Online Resource A. Study area and future landscape scenarios

## **Table A.1.** Regional differences between study areas.

| **Characteristics** | **Intensive region** | **Non-intensive region** |
| --- | --- | --- |
| Location | Huallaga River Valley | Huayabamba River Valley |
| Predominant land uses | Rice and papaya monocrops | Banana plantations and cacao agroforests |
| Main driver of deforestation | Industrial monocultures | Smallholder agriculture |
| Predominant forest type | Seasonally dry forest | Lowland tropical moist forests |
| Elevation | 265 - 358 m.a.s.l. | 330 - 436 m.a.s.l. |
| Plot size (ha) | 1.29 (±0.63 SD) | 1.29 (±0.67 SD) |
| Average annual temperature | 26 °C | 26 °C |
| Average annual precipitation | 1200–1400 | 1200–1400 |
| Precipitation seasonality | High | Low |
| Cacao farmers’ cooperative | Acopagro | Choba-choba |

## *A.1. Plot characteristics*

Plots were selected across gradients of landscape tree and cropland cover to represent the variation in agricultural intensity in each region (Table A.1). Plot elevations ranged from 256 to 358 m.a.s.l in the intensive region and 330 to 436 m.a.s.l in the non-intensive one. Plot sizes ranged from 0.3 to 2.3 ha, with an average (± SD) of 1.29 ± 0.63 ha in the intensive region and 1.29 ± 0.67 ha in the non-intensive one. All but three of the plots were separated by at least 500 m from the nearest plot (minimum distance = 407 m, average distance = 1093 m). Agroforestry systems belonged to smallholders enrolled in Acopagro (in the intensive region; https://alcacao.org/acopagro-ltda/) and Choba-choba (in the non-intensive region; https://www.chobachoba.com/en) farmer cooperatives, both of which maintain high organic standards.


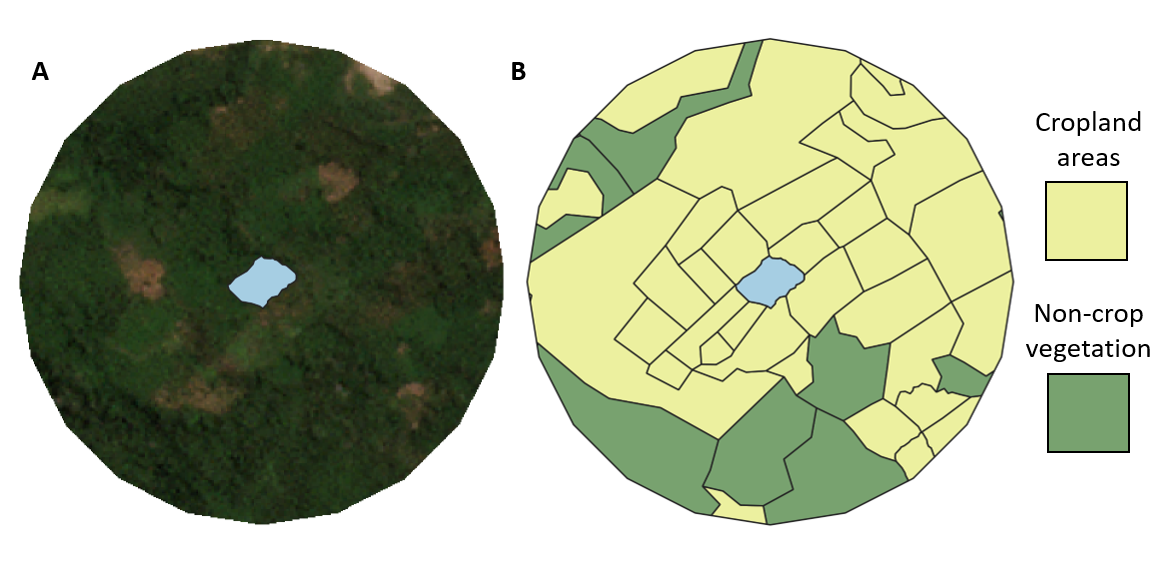


**Figure A.1.** Example manual classification of satellite images (A) used to estimate landscape cropland cover (B) in a 500-m radius buffer from the centroid of the plot. Cropland areas represent monocrops (e.g., orchards, cacao monocultures, banana plantations and corn fields), while non-crop vegetation represents mainly secondary forests. Satellite images were extracted from PlanetScope for September 2022.

*A.2. Workflow for the calculation of future landscape tree and cropland cover values under the Deforestation scenario (Fig. A.2.A):*

1. Extract landscape tree cover values in 250 and 500 m buffers for each plot using the raster layer from Brandt et al. (2023) and adjust the resolution from ca. 11.05 m to ca. 27.62 m to match the tree cover loss data from Hansen et al. (2013).
2. Calculate the total tree-covered area in the buffer multiplying the proportion of tree cover in each pixel (0 – 1) by its area (763 m^2^).
3. Extract tree cover loss data for the 2020 – 2023 period from Hansen et al. (2013).
4. Combining the two previous layers, calculate the area of tree cover lost each year.
5. Calculate the mean annual rate of tree cover loss in the 2020 – 2023 period and the remaining tree cover in 2024.
6. Calculate the future tree cover (year 2050) under the Deforestation scenario by sequentially subtracting the area of tree cover lost each year.
7. Use the manual classification of cropland areas based on satellite images to calculate the area of tree cover outside cropland areas (potential areas for cropland expansion).
8. Calculate future cropland cover assuming that cropland areas will expand to tree-covered non-cropland areas. If the area of tree cover outside croplands is larger than the area of tree cover lost during the 2020 – 2050 period, then the increase in cropland cover is similar to the area of tree cover lost. If the area of tree cover outside croplands is smaller than the area of tree cover lost during the 2020 – 2050 period, then the increase in cropland cover is similar to the area of tree cover outside croplands, as cropland cover cannot expand more than the available space.

*A.3. Workflow for the calculation of future landscape tree and cropland cover values under the Reforestation scenario (Fig. A.2.B):*

1. Extract landscape tree cover values in 250 and 500 m for the year 2000 from Hansen et al. (2013) and the tree cover data for 2020 adjusted from Brandt et al. (2023)
2. Calculate the percentage of tree cover lost from 2000 to 2020.
3. Calculate future tree and cropland cover under the Reforestation scenario. This scenario predicts the recovery of the tree cover lost in the 2000 – 2020 period through cropland reforestation (therefore reducing the cropland cover in the same amount).

*A.4. Estimating future edge density values based on future landscape tree cover (Fig. A.2.C):*

To estimate the future edge density, we calculated the relationship between tree cover and edge density for each spatial scale and region separately, fitting Generalized Additive Models (GAMs) in the R package *mgcv* (Wood, 2025). For this, we created 350 points distributed uniformly across each region (700 points in total) and extracted tree cover and each density values in 250 and 500-m buffers for each point. We then run four different GAMs to calculate how edge density values in 250 and 500-m buffers (response variables) varied depending on the percentage of tree cover (explanatory variable) in each region. GAMs were fitted using a Gaussian distribution and a nine-degree smoother, which was selected after comparing the deviance explained by candidate models fitted with different smoothers. Finally, we used the four different models (Fig. A.2.C) to predict future edge density values based on the future tree cover around each plot in each landscape scenario.


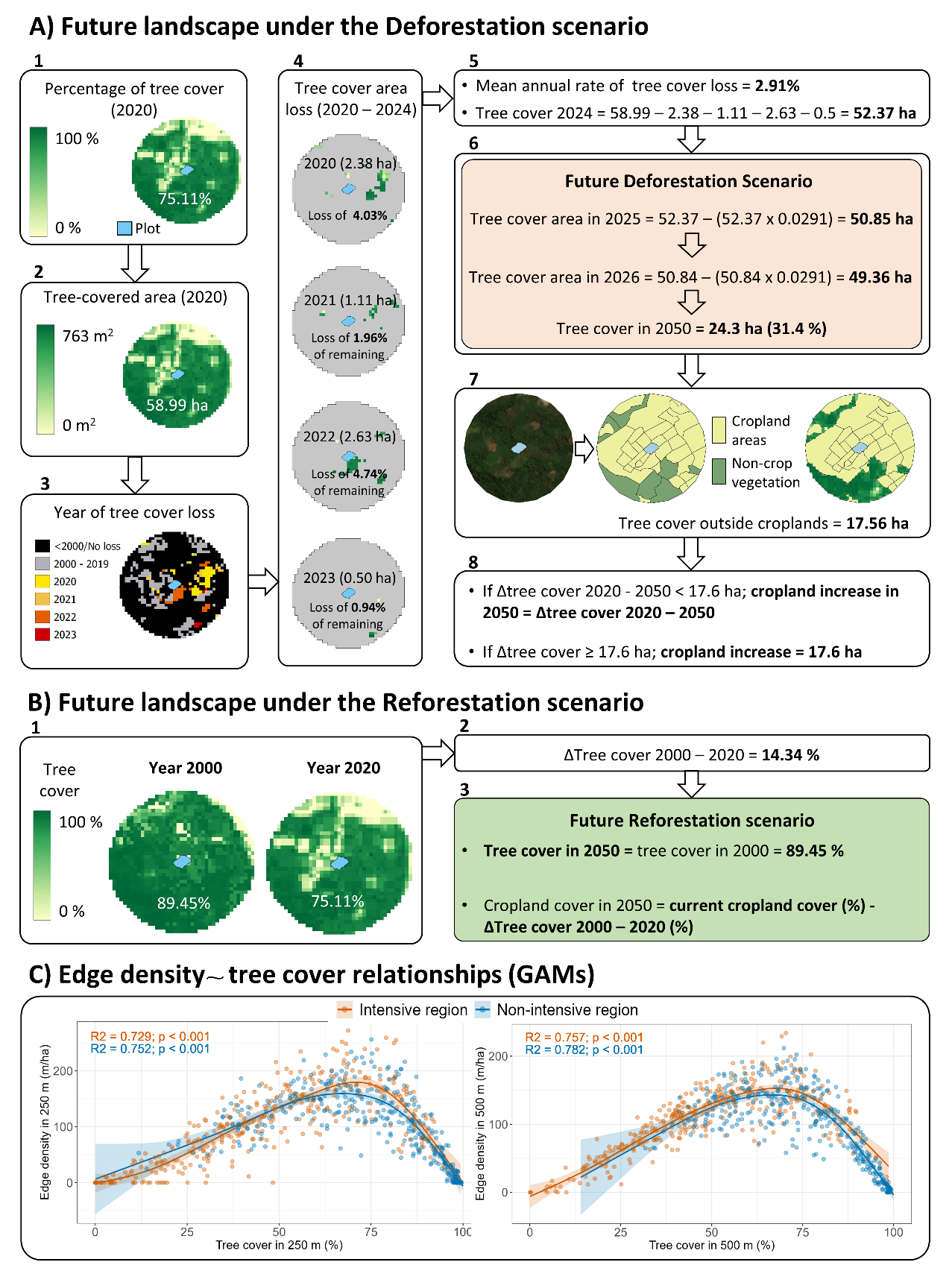


**Figure A.2**. Workflow used to calculate future tree and cropland cover in 2050 under the Deforestation (A) and Reforestation scenarios (B). The values of future edge density were predicted from the future tree cover values based on the relationship between both variables calculated with Generalized Additive Models (GAMs, C).

## **Table A.2.** Current and future values of landscape predictors under each scenario.

| **Scenario** | **Region** | **Buffer** | **Tree cover** | | | **Edge density** | | | **Cropland cover** | | |
| --- | --- | --- | --- | --- | --- | --- | --- | --- | --- | --- | --- |
|  |  |  | **Min** | **Max** | **Mean (SD)** | **Min** | **Max** | **Mean (SD)** | **Min** | **Max** | **Mean (SD)** |
| Current | Intensive | 250 m | 35.2 | 85.2 | 62.3 (18) | 103.8 | 227.6 | 169.9 (40.8) | 33.1 | 94.5 | 66.2 (18) |
|  |  | 500 m | 23 | 79.4 | 53.4 (19.3) | 59.8 | 230.3 | 142.8 (51.2) | 46.6 | 86.4 | 65.7 (14.3) |
|  | Non-intensive | 250 m | 42.5 | 94 | 73.2 (17.1) | 88.5 | 240.4 | 139.5 (41.3) | 19.2 | 92.9 | 55.2 (18.6) |
|  |  | 500 m | 42.6 | 89 | 68.3 (17.2) | 102.2 | 172.9 | 129.7 (20.7) | 39.4 | 73 | 49.5 (9.1) |
| Deforestation | Intensive | 250 m | 4.9 | 79.1 | 45.4 (21.9) | 5.1 | 176.6 | 114.8 (58.7) | 49.1 | 97.5 | 79 (17.5) |
|  |  | 500 m | 1.7 | 53.4 | 28.9 (16.6) | 0 | 137.3 | 73.3 (46.3) | 50.9 | 94.4 | 83.3 (10.8) |
|  | Non-intensive | 250 m | 15.5 | 95.3 | 55 (27.8) | 35.5 | 159 | 99.2 (37.3) | 24.7 | 99.6 | 65.7 (21.1) |
|  |  | 500 m | 25 | 80.6 | 47.4 (21.4) | 54.8 | 142.7 | 97.6 (33.2) | 49.1 | 95.7 | 65.5 (13.3) |
| Reforestation | Intensive | 250 m | 45.7 | 85.2 | 69.4 (10.6) | 122.3 | 179.5 | 163.6 (17) | 26.3 | 88.5 | 59 (20) |
|  |  | 500 m | 48.6 | 87.1 | 68.3 (11.1) | 100.1 | 152.1 | 140.3 (14.9) | 23.5 | 81.3 | 53.8 (13.5) |
|  | Non-intensive | 250 m | 56.9 | 98.5 | 86 (11.2) | 7.7 | 158.3 | 88.7 (47.8) | 9.8 | 78.3 | 42.3 (18) |
|  |  | 500 m | 65 | 97.3 | 82.2 (11.2) | 15.7 | 142.9 | 94.8 (49.6) | 16.2 | 59 | 35.6 (11.9) |

# Online Resource B. Functional and phylogenetic diversity analyses.

## *B.1. Descriptions of traits used in the calculation of aerial insectivore and frugivore functional diversity:*

**Forearm length and body mass (aerial insectivores and frugivores):** Forearm length and body mass are estimators of body size and relate to dispersal, home range size, energy requirements and extinction risk. Large-bodied species tend to have larger home ranges, energy requirements and dispersal ability, making them more vulnerable to habitat fragmentation than small species but also capable of searching for resources on a larger spatial scale (Colombo et al., 2023; Farneda et al., 2015; Núñez et al., 2019). Given that forearm length and body mass are shaped by the environment but also affect species interactions (e.g., larger frugivorous bats can disperse larger fruits; Sivault et al., 2023), they can be classified as both response and effect traits.

For aerial insectivores, we obtained values of forearm length and body mass from Conenna et al. (2021). For phyllostomids, we used the average forearm length and body mass of adult bats (excluding pregnant females) captured in the mist nets.

**Wing loading and aspect ratio (aerial insectivores and frugivores):** Both wing loading and aspect ratio are associated with bat flight ability. Species with high wing loading and aspect ratio can fly at high speeds, have limited maneuverability in cluttered environments, and are well adapted to foraging in open areas (Marinello & Bernard, 2014; Norberg et al., 1997). Wing loading and aspect ratio were defined as in (Norberg et al., 1997). Wing loading and aspect ratio also determine a species' ability to hunt or consume fruit in different environments, acting as both response and effect traits.

Values of wing loading and aspect ratio for both bat groups were obtained from Conenna et al. (2021). We estimated missing values using linear models. The wing loading of two aerial insectivore species (*Cynomops planirostris* and *Myotis riparius*) was estimated based on their biomass using a linear model (mode: wing loading ~ biomass; adjusted R^2^ = 0.643; *p* < 0.01) and values of aspect ratio of *Myotis riparius* based on its wing loading (model: aspect ratio ~ wing loading; adjusted R^2^ = 0.534; *p* < 0.001). Additionally, we estimated values of wing loading of one phyllostomid species (*Dermanura glauca*) based on its biomass (adjusted R^2^ = 0.668; p < 0.001) and its aspect ratio based on its wing loading (adjusted R^2^ = 0.263; p = 0.003).

**Preferred vertical stratum (aerial insectivores and frugivores):** Bat responses to changes in land use often vary according to their preferred vertical strata, with understory species generally being more sensitive to habitat alterations (García-Morales et al., 2013). Additionally, canopy and understory insectivorous and frugivorous bats prey on insects or consume fruits at different heights, affecting the resource vertical stratification. Therefore, the preferred vertical stratum is both a response and an effect functional trait.

We categorized aerial insectivores and frugivores into canopy or understory species following Marques et al. (2016) and Mas (2014). Additionally, we used data from Carvalho et al. (2021) and Rex et al. (2011) to complete missing trait information.

**Frequency of Maximum Energy (aerial insectivores only):** The Frequency of Maximum Energy (kHz) is the frequency at which the greatest call intensity is recorded (Lopez-Baucells et al., 2016). Low-frequency pulses travel farther, reduce echoes from nearby objects, and experience less attenuation, making them typical of species that forage in open habitats (Jones & Holderied, 2007). The Frequency of Maximum Energy reflects aerial insectivore responses to environmental conditions such as vegetation openness, but also determines in which environment the species can hunt. Therefore, it is both a response and an effect trait.

The Frequency of Maximum Energy of the registered aerial insectivore species was obtained from our recordings.

**Call shape (aerial insectivores only):** Call shape refers to the shape of bat echolocation calls in the spectrograms of the recordings, which vary depending on the habitat type and foraging mode of the species (Jones, 1999). Similarly to the Frequency of Maximum Energy, aerial insectivore call shape is influenced by the environment and determines the potential prey of the species, acting as both a response and an effect functional trait.

We classified call shapes of aerial insectivores into six categories following Arias-Aguilar et al. (2018) and Lopez-Baucells et al. (2016). 1) Constant frequency calls (CF) are characteristic of species foraging in cluttered environments; 2) constant frequency–frequency modulated calls (CF-FM), characteristic of species foraging in both cluttered and open environments; 3) constant frequency-frequency modulated-constant frequency calls (CF-FM-CF), characteristic of species foraging in forest edges and open environments; 4) frequency modulated calls (FM) characteristic of species foraging in cluttered or semi-cluttered environments; 5) frequency modulated-quasi-constant frequency calls (FM-QCF), characteristic of edge-foraging species; and 6) quasi-constant frequency calls (QCF), characteristic of species foraging in open and edge habitats (Colombo et al., 2023; Jones, 1999).

**Diet (frugivores only):** Diet composition serves as an indicator of different ecosystem services provided by bats, including biological pest control, seed dispersal and pollination. Additionally, frugivorous bats may respond differently to the environment depending on their degree of diet specialization, with more generalist species usually being less affected by disturbances (Bonaccorso et al., 1979). Therefore, diet is both a response and an effect trait.

We characterized the level of dietary specialization frugivores species using the percentage (%) contribution of invertebrates, fruits, and nectar to the species' diet, extracted from Wilman et al. (2014).

## *B.2. Functional diversity calculation:*

To include aerial insectivore sonotypes in the functional diversity analyses, we identified which of the species included in each sonoytpe had the highest chances of being recorded in our study based on habitat (Pacheco et al., 2021) and geographic distribution (IUCN, 2025), and used the functional traits of this species to represent each sonotype in the calculation of functional diversity.

We used the trait-by-species matrices to calculate the functional distance between species for each bat group separately using a corrected version of Gower´s distance that equalizes the contribution of correlated traits (Table 1) with the *gawdis* R package (Bello et al., 2021). The resulting distance matrices were used to estimate the functional richness (FRic) as the functional convex hull volume of the species assemblages captured/recorded in each plot and night with the *mFD* package (Magneville et al., 2022). We used null models to calculate the standardized effect size of the FRic (SES.FRic) and remove the influence of species richness on FRic (Mason et al., 2013). To calculate SES.FRic values, we created 999 null communities using the ‘independent swap’ algorithm of the R package *picante* (Kembel et al., 2020), which randomizes the species-by-night matrix maintaining the occurrence frequency of the species and the number of species detected each night. Species were randomized separately for each region to avoid including species occurring only in one region in the null communities of the other. Finally, we calculated the SES.FRic per sampling night and plot using the following formula:

$$SES.FRic= \frac{(observed FRic-mean null FRic)}{sd null FRic}$$

## **Table B.1.** Functional trait values of aerial insectivorous bats.

| **Species/sonotype** | **Most probable species** | **Forearm length** | **Biomass** | **Wing loading** | **Wing aspect ratio** | **Vertical strata** | **Frequency max. energy** | **Call shape** |
| --- | --- | --- | --- | --- | --- | --- | --- | --- |
| *Cormura brevirostris* |  | 46.5 | 9.3 | 8.2 | 7.2 | Canopy | 29.38 | QCF |
| *Cyttarops alecto* |  | 47.0 | 5.3 | 6.1 | 5.8 | Canopy | 36 | QCF |
| *Diclidurus albus* |  | 66.0 | 16.6 | 12.9 | 9.0 | Canopy | 24 | QCF |
| *Emballonuridae* II | *Centronycteris centralis* | 45.0 | 5.5 | 5.4 | 5.8 | Canopy | 42.71 | QCF |
| *Peropteryx kappleri* |  | 49.5 | 9.9 | 8.5 | 9.0 | Understory | 32 | QCF |
| *Peropteryx macrotis* |  | 43.5 | 5.7 | 4.9 | 5.8 | Canopy | 37.35 | QCF |
| *Rhynchonycteris naso ** |  | 38.1 | 4.1 | 4.3 | 6.5 | Understory | 89.69 | CF-FM |
| *Saccopteryx bilineata* |  | 46.0 | 8.1 | 5.9 | 6.1 | Canopy | 45.76 | QCF |
| *Saccopteryx leptura* |  | 39.0 | 4.7 | 3.3 | 7.1 | Canopy | 49.54 | QCF |
| *Molossidae* III | *Cynomops planirostris* | 31.5 | 12.8 | 10.0 | 9.0 | Canopy | 27.8 | QCF |
| *Molossops temminckii* |  | 28.5 | 5.9 | 9.4 | 7.5 | Canopy | 50.4 | QCF |
| *Molossus* II | *Molossus rufus* | 50.0 | 31.7 | 23.4 | 9.7 | Canopy | 26.5 | QCF |
| *Molossus molossus* |  | 38.2 | 13.7 | 16.0 | 8.7 | Canopy | 37.66 | QCF |
| *Nyctinomops macrotis* |  | 61.0 | 16.4 | 16.0 | 8.7 | Canopy | 17.5 | QCF |
| *Promops centralis* |  | 53.0 | 29.8 | 17.6 | 9.4 | Canopy | 26.03 | QCF |
| *Pteronotus gymnonotus* |  | 51.7 | 13.6 | 7.3 | 8.0 | Understory | 49 | CF-FM-CF |
| *Pteronotus fuscus* |  | 60.1 | 19.6 | 6.5 | 6.7 | Understory | 60.95 | CF |
| *Pteronotus personatus* |  | 44.5 | 8.0 | 7.9 | 5.8 | Understory | 65.45 | CF-FM-CF |
| *Noctilio albiventris* |  | 60.0 | 31.5 | 13.9 | 7.8 | Understory | 69.5 | CF-FM |
| *Noctilio leporinus ** |  | 79.4 | 29.9 | 15.2 | 9.0 | Understory | 35.27 | CF-FM |
| *Thyroptera* sp * | *Thyroptera tricolor* | 35.5 | 4.5 | 4.1 | 6.0 | Understory | 54.39 | FM |
| *Eptesicus* sp | *Eptesicus furinalis* | 39.2 | 7.7 | 7.3 | 6.2 | Canopy | 42.84 | FM-QCF |
| *Myotis nigricans* |  | 34.1 | 4.2 | 6.1 | 6.5 | Canopy | 56.2 | FM-QCF |
| *Myotis riparius* |  | 35.0 | 4.6 | 6.1 | 6.1 | Canopy | 57.68 | FM-QCF |
| *Vespertilionidae* I | *Lasiurus ega* | 47.5 | 12.2 | 11.9 | 5.8 | Canopy | 32.2 | FM-QCF |
| *Vespertilionidae* II | *Lasiurus blossevillii* | 41.7 | 13.0 | 11.9 | 5.8 | Canopy | 53.75 | FM-QCF |

Call shapes: CF = constant frequency; QCF = quasi-constant frequency; FM = frequency modulated. Most probable species were selected based on habitat specificity (Pacheco et al., 2021) and distribution (IUCN, 2025). See Table 2 for a list of references from which trait values were compiled. Species that were not included in the functional diversity analyses due to their low number of records are marked with * (see Table C.1).

## **Table B.2.** Functional trait values of frugivorous bats and diet composition of all phyllostomids captured.

| **Species** | **Forearm length** | **Biomass** | **Wing loading** | **Wing aspect ratio** | **Vertical strata** | **% vertebrates** | **% invertebrates** | **% fruits** | **% nectar** |
| --- | --- | --- | --- | --- | --- | --- | --- | --- | --- |
| *Anoura geoffroyi* | - | - | - | - | - | 0 | 30 | 30 | 40 |
| *Artibeus lituratus* | 71.7 | 74.9 | 17.7 | 6.1 | Canopy | 0 | 10 | 80 | 10 |
| *Artibeus obscurus* | 57.7 | 34.3 | 13.3 | 6.5 | Understory | 0 | 10 | 80 | 10 |
| *Artibeus planirostris* | 66.3 | 60.3 | 15.1 | 6.4 | Understory | 0 | 10 | 80 | 10 |
| *Carollia benkeithi* | 36.1 | 12.0 | 12.4 | 5.5 | Understory | 0 | 0 | 100 | 0 |
| *Carollia brevicauda* | 38.9 | 15.3 | 9.6 | 5.9 | Understory | 0 | 0 | 100 | 0 |
| *Carollia perspicillata* | 42.5 | 17.8 | 11.4 | 6.1 | Understory | 0 | 0 | 100 | 0 |
| *Chiroderma trinitatum* | 32.6 | 9.0 | 8.8 | 6.3 | Canopy | 0 | 0 | 100 | 0 |
| *Chiroderma villosum* | 44.3 | 22.0 | 14.0 | 6.4 | Canopy | 0 | 0 | 100 | 0 |
| *Dermanura glauca* | 36.5 | 10.3 | 10.0 | 6.1 | Canopy | 0 | 10 | 80 | 10 |
| *Desmodus rotundus* | - | - | - | - | - | 100 | 0 | 0 | 0 |
| *Glossophaga soricina* | - | - | - | - | - | 0 | 40 | 30 | 30 |
| *Hsunycteris thomasi* | - | - | - | - | - | 0 | 40 | 30 | 30 |
| *Lonchophylla robusta* | - | - | - | - | - | 0 | 40 | 30 | 30 |
| *Lophostoma brasiliense* | - | - | - | - | - | 0 | 90 | 10 | 0 |
| *Lophostoma carrikeri* | - | - | - | - | - | 0 | 90 | 10 | 0 |
| *Lophostoma silvicolum* | - | - | - | - | - | 0 | 90 | 10 | 0 |
| *Mesophylla macconnelli* | 35.8 | 10.0 | 7.5 | 5.8 | Understory | 0 | 10 | 90 | 0 |
| *Micronycteris minuta* | - | - | - | - | - | 0 | 80 | 20 | 0 |
| *Phyllostomus discolor* | - | - | - | - | - | 0 | 30 | 40 | 30 |
| *Phyllostomus elongatus* | - | - | - | - | - | 10 | 70 | 10 | 10 |
| *Phyllostomus hastatus* | - | - | - | - | - | 50 | 40 | 10 | 0 |
| *Rhinophylla pumilio* | 34.8 | 10.8 | 8.3 | 6.2 | Understory | 0 | 10 | 90 | 0 |
| *Sturnira giannae* | 43.1 | 20.8 | 12.2 | 6.5 | Understory | 0 | 0 | 100 | 0 |
| *Sturnira tildae* | 43.9 | 21.5 | 12.2 | 6.5 | Understory | 0 | 0 | 100 | 0 |
| *Trinycteris nicefori* | - | - | - | - | - | 0 | 80 | 20 | 0 |
| *Uroderma bilobatum* | 42.9 | 18.8 | 10.1 | 6.3 | Canopy | 0 | 10 | 80 | 10 |
| *Vampyriscus bidens* | 37.3 | 13.0 | 10.6 | 6.4 | Canopy | 0 | 0 | 100 | 0 |
| *Vampyrodes caraccioli* | 37.4 | 12.9 | 14.5 | 6.5 | Understory | 0 | 0 | 100 | 0 |

% refers to the percentage of the item in the species' diet. See Table 2 for a list of references from which trait values were compiled.

## *B.2. Phylogenetic diversity calculation:*

To estimate the phylogenetic diversity, we downloaded 1000 phylogenetic trees for the detected phyllostomid and aerial insectivore species from vertlife.org (Upham et al. 2019) and built a majority-rule consensus tree using the R package *ape* (Paradis et al., 2024). We used the mean pairwise phylogenetic distance (MPD) to measure the phylogenetic diversity of the bat assemblages. We calculated the standardized effect size of the MPD (SES.MPD) to remove the influence of species richness on the phylogenetic diversity using the R package *picante* (Kembel et al., 2020). We used the same 999 null assemblages created for the calculation of SES.FRic to calculate the SES.MPD values of the aerial insectivore and phyllostomid assemblages captured each night in each plot. Finally, we calculated the SES.MPD per sampling night and plot using the following formula:

$$SES.MPD= \frac{(observed MPD-mean null MPD)}{sd null MPD}$$

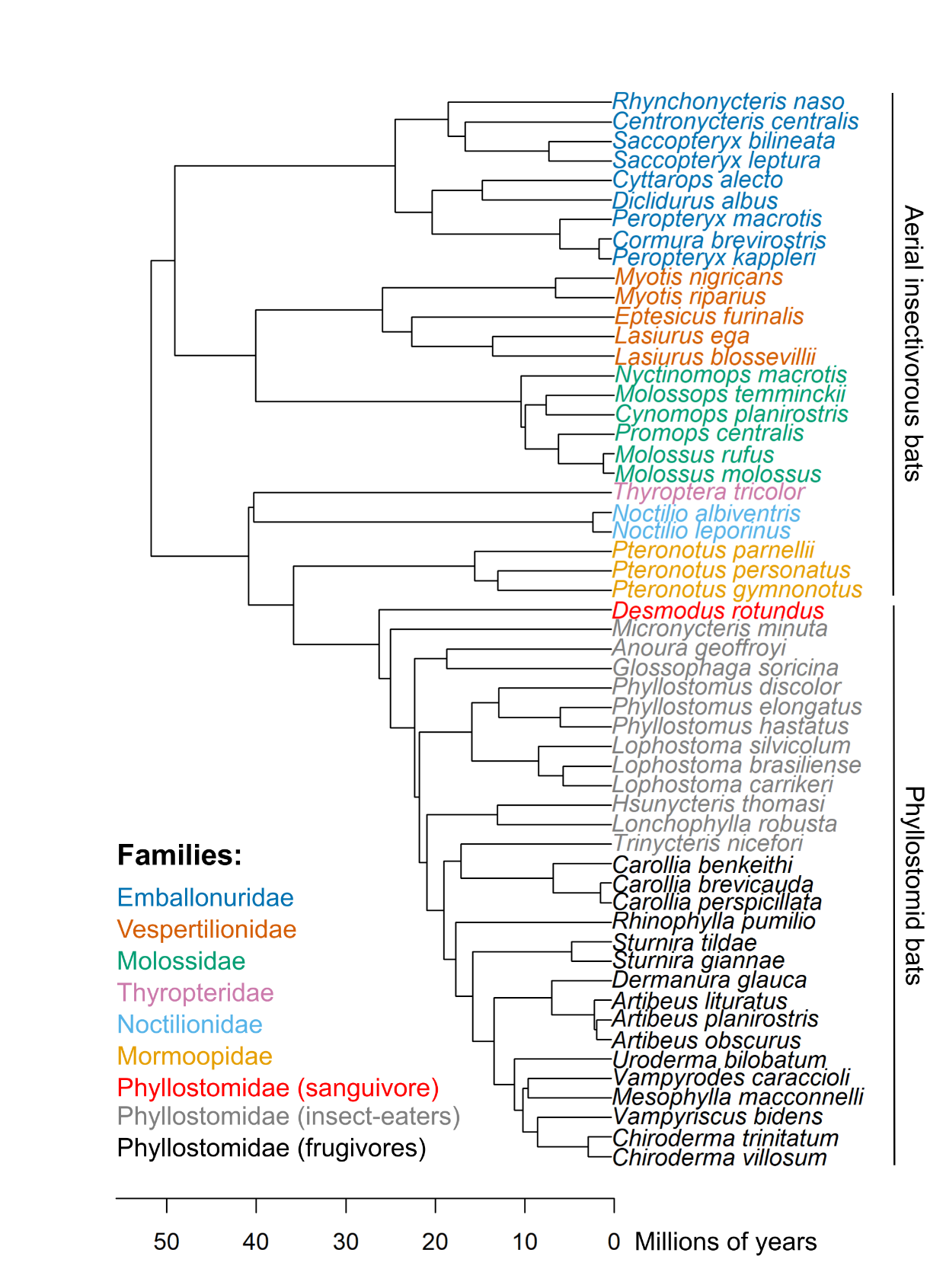


**Figure B.1.** Consensus phylogenetic tree used for the calculation of mean pairwise distances (MPD) between bat species, built from 1,000 trees extracted from Upham et al. (2019). Species are colored by family (all species) and guild (only phyllostomids).

*References Resources A and B:*

Arias-Aguilar, A., Hintze, F., Aguiar, L., Rufray, V., Bernard, E., & Ramos Pereira, M. J. (2018). Who’s calling? Acoustic identification of Brazilian bats. *Mammal Research*, *63*. https://doi.org/10.1007/s13364-018-0367-z

Bello, F. de, Botta-Dukat, Z., Leps, J., & Fibich, P. (2021). *gawdis: Multi-Trait Dissimilarity with more Uniform Contributions* (Version 0.1.3) [Computer software]. https://CRAN.R-project.org/package=gawdis

Brandt, J., Ertel, J., Spore, J., & Stolle, F. (2023). Wall-to-wall mapping of tree extent in the tropics with Sentinel-1 and Sentinel-2. *Remote Sensing of Environment*, *292*, 113574. https://doi.org/10.1016/j.rse.2023.113574

Carvalho, W. D., Mustin, K., Farneda, F. Z., de Castro, I. J., Hilário, R. R., Martins, A. C. M., Miguel, J. D., da SilvaXavier, B., & de Toledo, J. J. (2021). Taxonomic, functional and phylogenetic bat diversity decrease from more to less complex natural habitats in the Amazon. *Oecologia*, *197*(1), 223–239. https://doi.org/10.1007/s00442-021-05009-3

Colombo, G. T., Di Ponzio, R., Benchimol, M., Peres, C. A., & Bobrowiec, P. E. D. (2023). Functional diversity and trait filtering of insectivorous bats on forest islands created by an Amazonian mega dam. *Functional Ecology*, *37*(1), 99–111. https://doi.org/10.1111/1365-2435.14118

Conenna, I., Santini, L., Rocha, R., Monadjem, A., Cabeza, M., & Russo, D. (2021). Global patterns of functional trait variation along aridity gradients in bats. *Global Ecology and Biogeography*, *30*(5), 1014–1029. https://doi.org/10.1111/geb.13278

Farneda, F. Z., Rocha, R., López-Baucells, A., Groenenberg, M., Silva, I., Palmeirim, J. M., Bobrowiec, P. E. D., & Meyer, C. F. J. (2015). Trait-related responses to habitat fragmentation in Amazonian bats. *Journal of Applied Ecology*, *52*(5), 1381–1391. https://doi.org/10.1111/1365-2664.12490

García-Morales, R., Badano, E. I., & Moreno, C. E. (2013). Response of Neotropical Bat Assemblages to Human Land Use. *Conservation Biology*, *27*(5), 1096–1106. https://doi.org/10.1111/cobi.12099

Hansen, M. C., Potapov, P. V., Moore, R., Hancher, M., Turubanova, S. A., Tyukavina, A., Thau, D., Stehman, S. V., Goetz, S. J., Loveland, T. R., Kommareddy, A., Egorov, A., Chini, L., Justice, C. O., & Townshend, J. R. G. (2013). High-Resolution Global Maps of 21st-Century Forest Cover Change. *Science*, *342*(6160), 850–853. https://doi.org/10.1126/science.1244693

IUCN. (2025). In *The IUCN Red List of Threatened Species. Version 2025-1.* https://www.iucnredlist.org.

Jones, G. (1999). Scaling of echolocation call parameters in bats. *Journal of Experimental Biology*, *202*(23), 3359–3367. https://doi.org/10.1242/jeb.202.23.3359

Jones, G., & Holderied, M. W. (2007). Bat echolocation calls: Adaptation and convergent evolution. *Proceedings of the Royal Society B: Biological Sciences*, *274*(1612), 905–912. https://doi.org/10.1098/rspb.2006.0200

Kembel, S. W., Ackerly, D. D., Blomberg, S. P., Cornwell, W. K., Cowan, P. D., Helmus, M. R., Morlon, H., & Webb, C. O. (2020). *picante: Integrating Phylogenies and Ecology* (Version 1.8.2) [Computer software]. https://CRAN.R-project.org/package=picante

Lopez-Baucells, A., Rocha, R., Bobrowiec, P., Bernard, E., Palmeirim, J., & Meyer, C. (2016). *Field Guide to Amazonian Bats*. https://doi.org/10.13140/RG.2.2.23475.84003

Magneville, C., Loiseau, N., Albouy, C., Casajus, N., Claverie, T., Escalas, A., Leprieur, F., Maire, E., Mouillot, D., & Villéger, S. (2022). mFD: An R package to compute and illustrate the multiple facets of functional diversity. *Ecography*, *2022*(1). https://doi.org/10.1111/ecog.05904

Marinello, M. M., & Bernard, E. (2014). Wing morphology of Neotropical bats: A quantitative and qualitative analysis with implications for habitat use. *Canadian Journal of Zoology*, *92*(2), 141–147. https://doi.org/10.1139/cjz-2013-0127

Marques, J. T., Ramos Pereira, M. J., & Palmeirim, J. M. (2016). Patterns in the use of rainforest vertical space by Neotropical aerial insectivorous bats: All the action is up in the canopy. *Ecography*, *39*(5), 476–486. https://doi.org/10.1111/ecog.01453

Mas, M. (2014). *Vertical stratification on insectivorous bats ensembles in Central Amazon*.

Mason, N. W. H., de Bello, F., Mouillot, D., Pavoine, S., & Dray, S. (2013). A guide for using functional diversity indices to reveal changes in assembly processes along ecological gradients. *Journal of Vegetation Science*, *24*(5), 794–806. https://doi.org/10.1111/jvs.12013

Norberg, U. M., Rayner, J. M. V., & Lighthill, M. J. (1997). Ecological morphology and flight in bats (Mammalia; Chiroptera): Wing adaptations, flight performance, foraging strategy and echolocation. *Philosophical Transactions of the Royal Society of London. B, Biological Sciences*, *316*(1179), 335–427. https://doi.org/10.1098/rstb.1987.0030

Núñez, S. F., López-Baucells, A., Rocha, R., Farneda, F. Z., Bobrowiec, P. E. D., Palmeirim, J. M., & Meyer, C. F. J. (2019). Echolocation and Stratum Preference: Key Trait Correlates of Vulnerability of Insectivorous Bats to Tropical Forest Fragmentation. *Frontiers in Ecology and Evolution*, *7*. https://www.frontiersin.org/articles/10.3389/fevo.2019.00373

Pacheco, V., Diaz, S., Graham-Angeles, L., Flores-Quispe, M., Calizaya-Mamani, G., Ruelas, D., Sánchez-Vendizú, P., Pacheco, V., Diaz, S., Graham-Angeles, L., Flores-Quispe, M., Calizaya-Mamani, G., Ruelas, D., & Sánchez-Vendizú, P. (2021). Lista actualizada de la diversidad de los mamíferos del Perú y una propuesta para su actualización. *Revista Peruana de Biología*, *28*(4). https://doi.org/10.15381/rpb.v28i4.21019

Paradis, E., Blomberg, S., Bolker [aut, B., cph, Brown, J., Claramunt, S., Claude, J., Cuong, H. S., Desper, R., Didier, G., Durand, B., Dutheil, J., Ewing, R. J., Gascuel, O., Guillerme, T., Heibl, C., Ives, A., Jones, B., Krah [aut, F., … Vienne, D. de. (2024). *ape: Analyses of Phylogenetics and Evolution* (Version 5.8) [Computer software]. https://cran.r-project.org/web/packages/ape/index.html

Rex, K., Michener, R., Kunz, T. H., & Voigt, C. C. (2011). Vertical stratification of Neotropical leaf-nosed bats (Chiroptera: Phyllostomidae) revealed by stable carbon isotopes. *Journal of Tropical Ecology*, *27*(3), 211–222.

Sivault, E., McConkey, K. R., Bretagnolle, F., Sengupta, A., Lambert, J. E., Heymann, E. W., Herrel, A., & Forget, P.-M. (2023). Can body mass and skull morphology predict seed and fruit ingestion potential for mammal species? A test using extant species and its application to extinct species. *Functional Ecology*, *37*(5), 1504–1515. https://doi.org/10.1111/1365-2435.14300

Upham, N. S., Esselstyn, J. A., & Jetz, W. (2019). Inferring the mammal tree: Species-level sets of phylogenies for questions in ecology, evolution, and conservation. *PLOS Biology*, *17*(12), e3000494. https://doi.org/10.1371/journal.pbio.3000494

Wilman, H., Belmaker, J., Simpson, J., de la Rosa, C., Rivadeneira, M. M., & Jetz, W. (2014). EltonTraits 1.0: Species-level foraging attributes of the world’s birds and mammals. *Ecology*, *95*(7), 2027–2027. https://doi.org/10.1890/13-1917.1

Wood, S. (2025). *mgcv: Mixed GAM Computation Vehicle with Automatic Smoothness Estimation* (Version 1.9-3) [Computer software]. https://cran.r-project.org/web/packages/mgcv/index.html

# Online Resource C. Species lists and sample coverage evaluations.

## **Table C.1.** Number of passes and feeding buzzes (in parentheses) of aerial insectivorous bats recorded in each region.

| **Species/sonotype** | **Family** | **Intensive** | **Non-intensive** |
| --- | --- | --- | --- |
| *Cormura brevirostris* | Emballonuridae | 63 (0) | 661 (10) |
| *Cyttarops alecto* | Emballonuridae | 28 (9) | 0 (0) |
| *Diclidurus albus* | Emballonuridae | 47 (0) | 17 (0) |
| *Emballonuridae* II | Emballonuridae | 295 (0) | 8 (1) |
| *Eptesicus* sp. | Vespertilionidae | 420 (8) | 68 (2) |
| *Molossidae* III | Molossidae | 735 (35) | 646 (22) |
| *Molossops temminckii* | Molossidae | 28 (0) | 2 (0) |
| *Molossus* II | Molossidae | 1281 (84) | 1334 (78) |
| *Molossus molossus* | Molossidae | 2805 (241) | 534 (27) |
| *Myotis nigricans* | Vespertilionidae | 19772 (1514) | 5347 (234) |
| *Myotis riparius* | Vespertilionidae | 446 (13) | 55 (0) |
| *Noctilio albiventris* | Noctilionidae | 731 (30) | 47 (9) |
| *Noctilio leporinus* | Noctilionidae | 3 (0) | 0 (0) |
| *Nyctinomops macrotis* | Molossidae | 52 (2) | 84 (2) |
| *Peropteryx kappleri* | Emballonuridae | 81 (9) | 67 (6) |
| *Peropteryx macrotis* | Emballonuridae | 745 (154) | 21 (1) |
| *Promops centralis* | Molossidae | 460 (3) | 1757 (14) |
| *Pteronotus fuscus* | Mormoopidae | 2620 (71) | 3630 (50) |
| *Pteronotus gymnonotus* | Mormoopidae | 1135 (79) | 4827 (112) |
| *Pteronotus personatus* | Mormoopidae | 285 (9) | 103 (1) |
| *Rhynchonycteris naso* | Emballonuridae | 1 (0) | 0 (0) |
| *Saccopteryx bilineata* | Emballonuridae | 67 (7) | 695 (44) |
| *Saccopteryx leptura* | Emballonuridae | 1148 (101) | 659 (57) |
| *Thyroptera* sp. | Thyropteridae | 0 (0) | 2 (0) |
| *Vespertilionidae* I | Vespertilionidae | 39 (1) | 233 (1) |
| *Vespertilionidae* II | Vespertilionidae | 13 (0) | 14 (0) |

*Noctilio leporinus, Rhynchonycteris naso* and *Thyroptera* sp. were never recorded more than three times in one night and were excluded from the taxonomic, functional and phylogenetic analyses to avoid overrepresenting species that might have been recorded while commuting through the plot or adjacent areas.

## **Table C.2**. Number of captures of phyllostomid bats (family Phyllostomidae) in each region.

| **Species** | **Subfamily** | **Intensive** | **Non-intensive** |
| --- | --- | --- | --- |
| *Anoura geoffroyi* | Glossophaginae | 0 | 1 |
| *Artibeus lituratus* | Stenodermatinae | 148 | 180 |
| *Artibeus obscurus* | Stenodermatinae | 1 | 8 |
| *Artibeus planirostris* | Stenodermatinae | 111 | 47 |
| *Carollia benkeithi* | Carolliinae | 12 | 35 |
| *Carollia brevicauda* | Carolliinae | 2 | 7 |
| *Carollia perspicillata* | Carolliinae | 105 | 401 |
| *Chiroderma trinitatum* | Stenodermatinae | 0 | 1 |
| *Chiroderma villosum* | Stenodermatinae | 0 | 1 |
| *Dermanura glauca* | Stenodermatinae | 7 | 28 |
| *Desmodus rotundus* | Desmodontinae | 0 | 4 |
| *Glossophaga soricina* | Glossophaginae | 10 | 12 |
| *Hsunycteris thomasi* | Lonchophyllinae | 0 | 8 |
| *Lonchophylla robusta* | Lonchophyllinae | 1 | 19 |
| *Lophostoma brasiliense* | Phyllostominae | 2 | 0 |
| *Lophostoma carrikeri* | Phyllostominae | 1 | 0 |
| *Lophostoma silvicolum* | Phyllostominae | 0 | 1 |
| *Mesophylla macconnelli* | Stenodermatinae | 0 | 1 |
| *Micronycteris minuta* | Micronycterinae | 1 | 0 |
| *Phyllostomus discolor* | Phyllostominae | 136 | 32 |
| *Phyllostomus elongatus* | Phyllostominae | 0 | 2 |
| *Phyllostomus hastatus* | Phyllostominae | 55 | 24 |
| *Rhinophylla pumilio* | Carolliinae | 3 | 79 |
| *Sturnira giannae* | Stenodermatinae | 14 | 23 |
| *Sturnira tildae* | Stenodermatinae | 5 | 1 |
| *Trinycteris nicefori* | Glyphonycterinae | 0 | 1 |
| *Uroderma bilobatum* | Stenodermatinae | 41 | 65 |
| *Vampyriscus bidens* | Stenodermatinae | 0 | 1 |
| *Vampyrodes caraccioli* | Stenodermatinae | 3 | 5 |


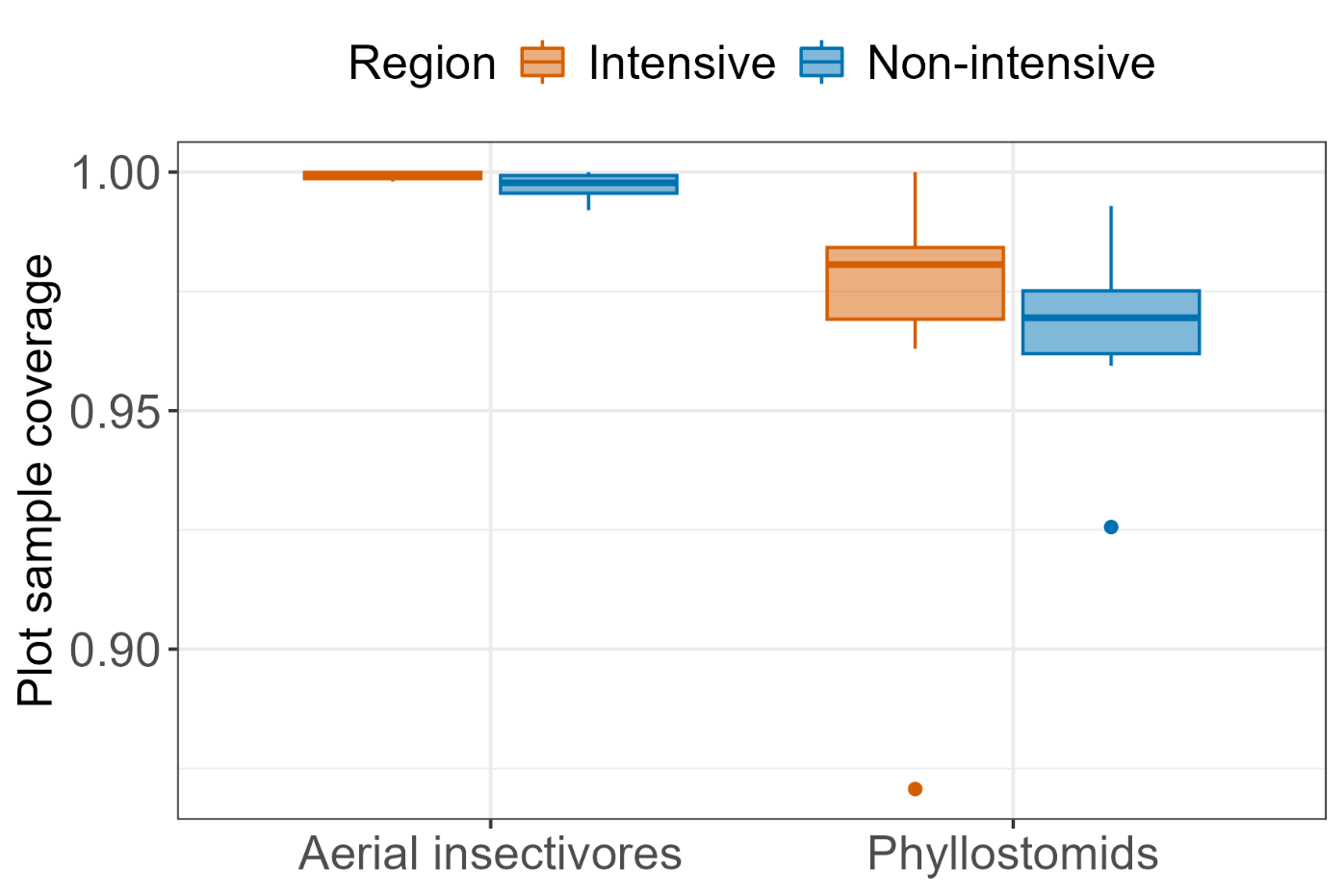


## Figure C.1**.** Plot-level sample coverage values of aerial insectivore and phyllostomid assemblages in the intensive and non-intensive regions

# Online Resource D. Model selection and results.

## **Table D.1.** AICc-based comparison of models including the landscape predictors measured at 250 and 500 m. Models with lower AICc (∆AICc > 2) are marked in bold.

| **Bat group** | **Response variable** | **250 m** | | **500 m** | | **∆AICc** | **Best buffer** |
| --- | --- | --- | --- | --- | --- | --- | --- |
|  |  | **AICc** | **R2 (marg.\|cond.)** | **AICc** | **R2 (marg.\|cond.)** |  |  |
| **Aerial insectivorous bats** | Richness | 559.31 | 0.13 \| 0.43 | 553.69 | 0.19 \| 0.42 | 5.63 | 500 m |
|  | Activity | 1520.24 | 0.22 \| 0.9 | 1514.04 | 0.31 \| 0.9 | 6.20 | 500 m |
|  | Simpson diversity | 381.20 | 0.06 \| 0.74 | 382.84 | 0.03 \| 0.74 | 1.64 | - |
|  | SES.FRic | 265.45 | 0.13 \| 0.54 | 266.85 | 0.11 \| 0.55 | 1.40 | - |
|  | SES.MPD | 305.82 | 0.32 \| 0.51 | 314.06 | 0.25 \| 0.52 | 8.24 | 250 m |
|  | Feeding activity (all) | 940.95 | 0.26 \| 0.85 | 928.19 | 0.4 \| 0.85 | 12.76 | 500 m |
|  | Feeding activity (understory spp.) | 544.97 | 0.24 \| 0.85 | 542.89 | 0.29 \| 0.85 | 2.08 | 500 m |
|  | Feeding activity (canopy spp.) | 909.58 | 0.3 \| 0.84 | 899.77 | 0.4 \| 0.83 | 9.81 | 500 m |
| **Phyllostomids** | Richness | 275.76 | 0.2 \| 0.2 | 275.34 | 0.21 \| 0.21 | 0.42 | - |
|  | Abundance | 486.26 | 0.4 \| 0.4 | 488.15 | 0.38 \| 0.39 | 1.88 | - |
|  | Simpson diversity | 187.31 | 0.16 \| 0.16 | 188.39 | 0.15 \| 0.15 | 1.08 | - |
|  | SES.FRic | 182.20 | 0.1 \| 0.28 | 176.84 | 0.17 \| 0.17 | 5.36 | 500 m |
|  | SES.MPD | 207.68 | 0.06 \| 0.06 | 207.55 | 0.06 \| 0.06 | 0.12 | - |
|  | Insect-eating phyll. abundance | 319.62 | 0.45 \| 0.45 | 341.96 | 0.12 \| 0.55 | 22.34 | 250 m |
|  | Small fruit-eating phyll. abundance | 413.07 | 0.66 \| 0.7 | 408.69 | 0.7 \| 0.7 | 4.38 | 500 m |
|  | Large fruit-eating phyll. abundance | 401.65 | 0.14 \| 0.14 | 404.89 | 0.1 \| 0.1 | 3.24 | 250 m |

## **Table D.2.1.** Anova tables (Type II Wald chisquare tests) of models analyzing landscape effects on aerial insectivore species richness, activity, Simpson diversity and functional diversity. Significant effects (*p* < 0.05) are marked in **bold**.

| **Response variable** | **Effect** | **Chisq** | **Df** | **p-value** |
| --- | --- | --- | --- | --- |
| Aerial insectivore species richness | Edge_density_500m | 0.5538 | 1 | 0.4568 |
|  | Cropland_500m | 0.0322 | 1 | 0.8576 |
|  | Tree_cover_500m | 2.3507 | 1 | 0.1252 |
|  | *Region* | *3.5588* | *1* | *0.0592* |
|  | Region:Edge_density_500m | 0.0739 | 1 | 0.7858 |
|  | Region:Cropland_500m | 2.0697 | 1 | 0.1502 |
|  | Region:Tree_cover_500m | 0.6270 | 1 | 0.4285 |
| Aerial insectivore activity | Edge_density_500m | 0.4342 | 1 | 0.5099 |
|  | Cropland_500m | 1.2212 | 1 | 0.2691 |
|  | Tree_cover_500m | 1.6084 | 1 | 0.2047 |
|  | **Region** | **5.5088** | **1** | **0.0189** |
|  | Region:Edge_density_500m | 0.4619 | 1 | 0.4967 |
|  | **Region:Cropland_500m** | **6.6724** | **1** | **0.0098** |
|  | Region:Tree_cover_500m | 2.3408 | 1 | 0.1260 |
| Aerial insectivore Simpson diversity | Edge_density_500m | 0.0202 | 1 | 0.8871 |
|  | Cropland_500m | 0.2440 | 1 | 0.6213 |
|  | Tree_cover_500m | 0.0009 | 1 | 0.9767 |
|  | Region | 0.0575 | 1 | 0.8104 |
|  | Region:Edge_density_500m | 0.5686 | 1 | 0.4508 |
|  | Region:Cropland_500m | 0.5301 | 1 | 0.4665 |
|  | Region:Tree_cover_500m | 0.0045 | 1 | 0.9466 |
| Aerial insectivore functional diversity (SES.FRic) | **Edge_density_500m** | **4.4037** | **1** | **0.0359** |
|  | Cropland_500m | 0.5515 | 1 | 0.4577 |
|  | Tree_cover_500m | 1.9049 | 1 | 0.1675 |
|  | Region | 0.0645 | 1 | 0.7995 |
|  | Region:Edge_density_500m | 0.9270 | 1 | 0.3357 |
|  | Region:Cropland_500m | 0.0075 | 1 | 0.9312 |
|  | **Region:Tree_cover_500m** | **4.5963** | **1** | **0.0320** |

## **Table D.2.2.** Anova tables (Type II Wald chisquare tests) of models analyzing landscape effects on aerial insectivore phylogenetic diversity and the number of feeding buzzes of all aerial insectivores and understory and canopy species separately. Significant effects (*p* < 0.05) are marked in **bold**.

| **Response variable** | **Effect** | **Chisq** | **Df** | **p-value** |
| --- | --- | --- | --- | --- |
| Aerial insectivore phylogenetic diversity (SES.MPD) | Edge_density_250m | 1.0172 | 1 | 0.3132 |
|  | Cropland_250m | 0.3511 | 1 | 0.5535 |
|  | **Tree_cover_250m** | **24.1838** | **1** | **< 0.0001** |
|  | **Region** | **5.9989** | **1** | **0.0143** |
|  | Region:Edge_density_250m | 1.2069 | 1 | 0.2720 |
|  | Region:Cropland_250m | 0.8995 | 1 | 0.3429 |
|  | **Region:Tree_cover_250m** | **7.8396** | **1** | **0.0051** |
| Aerial insectivore feeding buzzes (all spp.) | Edge_density_500m | 1.7216 | 1 | 0.1895 |
|  | Cropland_500m | 0.1750 | 1 | 0.6757 |
|  | Tree_cover_500m | 1.2583 | 1 | 0.2620 |
|  | **Region** | **14.3643** | **1** | **0.0002** |
|  | Region:Edge_density_500m | 0.0313 | 1 | 0.8596 |
|  | **Region:Cropland_500m** | **6.4291** | **1** | **0.0112** |
|  | Region:Tree_cover_500m | 1.4852 | 1 | 0.2230 |
| Aerial insectivore feeding buzzes (understory spp.) | Edge_density_500m | 0.3311 | 1 | 0.5650 |
|  | Cropland_500m | 0.0297 | 1 | 0.8632 |
|  | **Tree_cover_500m** | **6.7175** | **1** | **0.0095** |
|  | Region | 0.1426 | 1 | 0.7057 |
|  | Region:Edge_density_500m | 0.3889 | 1 | 0.5329 |
|  | **Region:Cropland_500m** | **4.2855** | **1** | **0.0384** |
|  | Region:Tree_cover_500m | 1.4848 | 1 | 0.2230 |
| Aerial insectivore feeding buzzes (canopy spp.) | Edge_density_500m | 2.2921 | 1 | 0.1300 |
|  | Cropland_500m | 0.0225 | 1 | 0.8808 |
|  | Tree_cover_500m | 0.0701 | 1 | 0.7913 |
|  | **Region** | **17.9608** | **1** | **0.0000** |
|  | Region:Edge_density_500m | 0.0379 | 1 | 0.8457 |
|  | *Region:Cropland_500m* | *3.7477* | *1* | *0.0529* |
|  | Region:Tree_cover_500m | 0.6344 | 1 | 0.4257 |

## **Table D.2.3.** Anova tables (Type II Wald chisquare tests) of models analyzing landscape effects on the species richness, abundance, Simpson diversity and functional diversity of the overall frugivore assemblage. Significant effects (*p* < 0.05) are marked in **bold**.

| **Response variable** | **Effect** | **Chisq** | **Df** | **p-value** |
| --- | --- | --- | --- | --- |
| Frugivore species richness | Edge_density_500m | 0.0177 | 1 | 0.8942 |
|  | Cropland_500m | 1.2259 | 1 | 0.2682 |
|  | Tree_cover_500m | 0.0000 | 1 | 0.9998 |
|  | *Region* | *3.8287* | *1* | *0.0504* |
|  | Region:Edge_density_500m | 0.0463 | 1 | 0.8297 |
|  | Region:Cropland_500m | 0.1383 | 1 | 0.7100 |
|  | Region:Tree_cover_500m | 0.0075 | 1 | 0.9309 |
| Frugivore abundance | Edge_density_500m | 0.1827 | 1 | 0.6690 |
|  | Cropland_500m | 1.5083 | 1 | 0.2194 |
|  | **Tree_cover_500m** | **4.8925** | **1** | **0.0270** |
|  | **Region** | **14.3260** | **1** | **0.0002** |
|  | Region:Edge_density_500m | 0.4958 | 1 | 0.4814 |
|  | *Region:Cropland_500m* | *3.5499* | *1* | *0.0595* |
|  | Region:Tree_cover_500m | 0.2906 | 1 | 0.5899 |
| Frugivore Simpson diversity | Edge_density_500m | 0.1348 | 1 | 0.7135 |
|  | Cropland_500m | 1.5261 | 1 | 0.2167 |
|  | **Tree_cover_500m** | **4.5897** | **1** | **0.0322** |
|  | Region | 0.6725 | 1 | 0.4122 |
|  | Region:Edge_density_500m | 0.3025 | 1 | 0.5823 |
|  | Region:Cropland_500m | 0.1373 | 1 | 0.7109 |
|  | Region:Tree_cover_500m | 0.2934 | 1 | 0.5881 |
| Frugivore functional diversity (SES.FRic) | Edge_density_500m | 0.6295 | 1 | 0.4275 |
|  | Cropland_500m | 0.0955 | 1 | 0.7573 |
|  | **Tree_cover_500m** | **6.6810** | **1** | **0.0097** |
|  | Region | 1.7887 | 1 | 0.1811 |
|  | Region:Edge_density_500m | 0.2421 | 1 | 0.6227 |
|  | *Region:Cropland_500m* | *2.9245* | *1* | *0.0872* |
|  | Region:Tree_cover_500m | 0.4495 | 1 | 0.5026 |

## **Table D.2.4.** Anova tables (Type II Wald chisquare tests) of models analyzing landscape effects on the phylogenetic diversity of the overall frugivore assemblage and the abundance of insect-eating phyllostomids, and small- and large-sized frugivorous species. Significant effects (*p* < 0.05) are marked in **bold**.

| **Response variable** | **Effect** | **Chisq** | **Df** | **p-value** |
| --- | --- | --- | --- | --- |
| Frugivore phylogenetic diversity (SES.MPD) | Edge_density_500m | 0.0001 | 1 | 0.9936 |
|  | Cropland_500m | 0.0719 | 1 | 0.7886 |
|  | Tree_cover_500m | 0.9583 | 1 | 0.3276 |
|  | Region | 0.0066 | 1 | 0.9351 |
|  | Region:Edge_density_500m | 0.0034 | 1 | 0.9536 |
|  | Region:Cropland_500m | 1.3871 | 1 | 0.2389 |
|  | Region:Tree_cover_500m | 0.0357 | 1 | 0.8502 |
| Insect-eating phyllostomid abundance | **Edge_density_250m** | **35.1189** | **1** | **0.0000** |
|  | **Cropland_250m** | **5.6640** | **1** | **0.0173** |
|  | **Tree_cover_250m** | **6.0520** | **1** | **0.0139** |
|  | Region | 1.4258 | 1 | 0.2325 |
|  | Region:Edge_density_250m | 1.1495 | 1 | 0.2837 |
|  | Region:Cropland_250m | 2.2813 | 1 | 0.1309 |
|  | *Region:Tree_cover_250m* | *3.4025* | *1* | *0.0651* |
| Small frugivore abundance | Edge_density_500m | 0.9603 | 1 | 0.3271 |
|  | Cropland_500m | 0.7813 | 1 | 0.3767 |
|  | *Tree_cover_500m* | *3.7481* | *1* | *0.0529* |
|  | **Region** | **60.0033** | **1** | **0.0000** |
|  | Region:Edge_density_500m | 1.6559 | 1 | 0.1982 |
|  | **Region:Cropland_500m** | **7.0680** | **1** | **0.0078** |
|  | *Region:Tree_cover_500m* | *3.6801* | *1* | *0.0551* |
| Large frugivore abundance | Edge_density_250m | 2.4937 | 1 | 0.1143 |
|  | Cropland_250m | 0.6163 | 1 | 0.4324 |
|  | *Tree_cover_250m* | *3.6492* | *1* | *0.0561* |
|  | Region | 1.2859 | 1 | 0.2568 |
|  | Region:Edge_density_250m | 0.9055 | 1 | 0.3413 |
|  | Region:Cropland_250m | 1.1049 | 1 | 0.2932 |
|  | Region:Tree_cover_250m | 1.0370 | 1 | 0.3085 |

## **Table D.3.** Summary of GLMMs analyzing the responses of aerial insectivorous bats and phyllostomids to landscape predictors.

**Table D.3.1.** Aerial insectivore species richness.

| **Aerial insectivore species richness** | | | |
| --- | --- | --- | --- |
| **Predictors** | **Log-Mean** | **CI** | **p** |
| (Intercept) | 2.17 | 1.98 – 2.36 | <0.001 |
| Edge_density_500m | -0.04 | -0.22 – 0.13 | 0.614 |
| Cropland_cover_500m | 0.05 | -0.11 – 0.22 | 0.526 |
| Tree_cover_500m | -0.02 | -0.28 – 0.23 | 0.852 |
| Region [Non-intensive] | -0.31 | -0.59 – -0.03 | 0.031 |
| Region [Non-intensive]: Edge_density_500m | -0.05 | -0.39 – 0.30 | 0.786 |
| Region [Non-intensive]: Cropland_cover_500m | -0.23 | -0.54 – 0.08 | 0.15 |
| Region [Non-intensive]: Tree_cover_500m | -0.12 | -0.43 – 0.18 | 0.428 |
| Random Effects | | | |
| σ2 | 0.11 |  |  |
| τ00 Plot | 0.03 |  |  |
| τ00 Plot_round | 0.02 |  |  |

**Table D.3.2.** Aerial insectivore activity.

| **Aerial insectivore activity** | | | |
| --- | --- | --- | --- |
| **Predictors** | **Log-Mean** | **CI** | **p** |
| (Intercept) | 6.19 | 5.71 – 6.67 | <0.001 |
| Edge_density_500m | -0.21 | -0.65 – 0.24 | 0.36 |
| Cropland_cover_500m | 0.13 | -0.30 – 0.57 | 0.547 |
| Tree_cover_500m | 0.2 | -0.45 – 0.84 | 0.552 |
| Region [Non-intensive] | -1.02 | -1.70 – -0.33 | 0.004 |
| Region [Non-intensive]: Edge_density_500m | 0.3 | -0.56 – 1.16 | 0.497 |
| Region [Non-intensive]: Cropland_cover_500m | -0.99 | -1.75 – -0.24 | 0.01 |
| Region [Non-intensive]: Tree_cover_500m | -0.6 | -1.37 – 0.17 | 0.126 |
| Random Effects | | | |
| σ2 | 0.09 |  |  |
| τ00 Plot | 0.27 |  |  |
| τ00 Plot_round | 0.3 |  |  |

**Table D.3.3.** Aerial insectivore Simpson diversity.

| **Aerial insectivore Simpson diversity** | | | |
| --- | --- | --- | --- |
| **Predictors** | **Estimates** | **CI** | **p** |
| (Intercept) | 2.94 | 2.10 – 3.78 | <0.001 |
| Edge_density_500m | 0.2 | -0.58 – 0.99 | 0.61 |
| Cropland_cover_500m | 0.32 | -0.45 – 1.09 | 0.409 |
| Tree_cover_500m | 0.02 | -1.11 – 1.16 | 0.968 |
| Region [Non-intensive] | 0.14 | -1.06 – 1.34 | 0.817 |
| Region [Non-intensive]: Edge_density_500m | -0.58 | -2.10 – 0.93 | 0.451 |
| Region [Non-intensive]: Cropland_cover_500m | -0.49 | -1.81 – 0.83 | 0.467 |
| Region [Non-intensive]: Tree_cover_500m | -0.05 | -1.40 – 1.31 | 0.947 |
| Random Effects | | | |
| σ2 | 0.63 |  |  |
| τ00 Plot | 0.71 |  |  |
| τ00 Plot_round | 1.03 |  |  |

**Table D.3.4.** Aerial insectivore functional diversity (SES.FRic).

| **Aerial insectivore functional diversity (SES.FRic)** | | | |
| --- | --- | --- | --- |
| **Predictors** | **Estimates** | **CI** | **p** |
| (Intercept) | 0.04 | -0.32 – 0.40 | 0.83 |
| Edge_density_500m | -0.22 | -0.56 – 0.11 | 0.195 |
| Cropland_cover_500m | 0.09 | -0.23 – 0.42 | 0.575 |
| Tree_cover_500m | 0.26 | -0.23 – 0.74 | 0.297 |
| Region [Non-intensive] | 0.06 | -0.46 – 0.57 | 0.83 |
| Region [Non-intensive]: Edge_density_500m | -0.32 | -0.97 – 0.33 | 0.336 |
| Region [Non-intensive]: Cropland_cover_500m | 0.03 | -0.55 – 0.60 | 0.931 |
| Region [Non-intensive]: Tree_cover_500m | -0.63 | -1.21 – -0.05 | 0.032 |
| Random Effects | | | |
| σ2 | 0.34 |  |  |
| τ00 Plot | 0 |  |  |
| τ00 Plot_round | 0.33 |  |  |

**Table D.3.5.** Aerial insectivore phylogenetic diversity (SES.MPD).

| **Aerial insectivore phylogenetic diversity (SES.MPD)** | | | |
| --- | --- | --- | --- |
| **Predictors** | **Estimates** | **CI** | **p** |
| (Intercept) | 0.07 | -0.26 – 0.40 | 0.672 |
| Edge_density_250m | -0.25 | -0.58 – 0.08 | 0.136 |
| Cropland_cover_250m | 0.18 | -0.14 – 0.50 | 0.276 |
| Tree_cover_250m | 0.23 | -0.11 – 0.57 | 0.185 |
| Region [Non-intensive] | -0.52 | -0.97 – -0.07 | 0.025 |
| Region [Non-intensive]: Edge_density_250m | 0.26 | -0.21 – 0.73 | 0.272 |
| Region [Non-intensive]: Cropland_cover_250m | -0.22 | -0.68 – 0.24 | 0.343 |
| Region [Non-intensive]: Tree_cover_250m | 0.67 | 0.20 – 1.13 | 0.005 |
| Random Effects | | | |
| σ2 | 0.59 |  |  |
| τ00 Plot | 0.02 |  |  |
| τ00 Plot_round | 0.22 |  |  |

**Table D.3.6.** Aerial insectivore feeding buzzes (all species).

| **Aerial insectivore feeding buzzes (all spp.)** | | | |
| --- | --- | --- | --- |
| **Predictors** | **Log-Mean** | **CI** | **p** |
| (Intercept) | 3.49 | 3.02 – 3.96 | <0.001 |
| Edge_density_500m | -0.27 | -0.71 – 0.17 | 0.223 |
| Cropland_cover_500m | 0.24 | -0.19 – 0.67 | 0.281 |
| Tree_cover_500m | 0.13 | -0.50 – 0.76 | 0.688 |
| Region [Non-intensive] | -1.5 | -2.20 – -0.81 | <0.001 |
| Region [Non-intensive]: Edge_density_500m | 0.08 | -0.77 – 0.93 | 0.86 |
| Region [Non-intensive]: Cropland_cover_500m | -1 | -1.77 – -0.23 | 0.011 |
| Region [Non-intensive]: Tree_cover_500m | -0.47 | -1.23 – 0.29 | 0.223 |
| Random Effects | | | |
| σ2 | 0.2 |  |  |
| τ00 Plot | 0.09 |  |  |
| τ00 Plot_round | 0.56 |  |  |

**Table D.3.7.** Aerial insectivore feeding buzzes (understory species).

| **Aerial insectivore feeding buzzes (understory spp.)** | | | |
| --- | --- | --- | --- |
| **Predictors** | **Log-Mean** | **CI** | **p** |
| (Intercept) | 0.82 | 0.17 – 1.46 | 0.013 |
| Edge_density_500m | 0.24 | -0.34 – 0.81 | 0.415 |
| Cropland_cover_500m | 0.27 | -0.30 – 0.85 | 0.353 |
| Tree_cover_500m | -0.18 | -1.02 – 0.66 | 0.677 |
| Region [Non-intensive] | -0.49 | -1.47 – 0.48 | 0.323 |
| Region [Non-intensive]: Edge_density_500m | -0.36 | -1.48 – 0.77 | 0.533 |
| Region [Non-intensive]: Cropland_cover_500m | -1.16 | -2.27 – -0.06 | 0.038 |
| Region [Non-intensive]: Tree_cover_500m | -0.63 | -1.64 – 0.38 | 0.223 |
| Random Effects | | | |
| σ2 | 0.22 |  |  |
| τ00 Plot | 0.39 |  |  |
| τ00 Plot_round | 0.51 |  |  |

**Table D.3.8.** Aerial insectivore feeding buzzes (canopy species).

| **Aerial insectivore feeding buzzes (canopy spp.)** | | | |
| --- | --- | --- | --- |
| **Predictors** | **Log-Mean** | **CI** | **p** |
| (Intercept) | 3.41 | 2.91 – 3.91 | <0.001 |
| Edge_density_500m | -0.33 | -0.79 – 0.13 | 0.161 |
| Cropland_cover_500m | 0.23 | -0.23 – 0.68 | 0.336 |
| Tree_cover_500m | 0.18 | -0.49 – 0.85 | 0.605 |
| Region [Non-intensive] | -1.69 | -2.42 – -0.96 | <0.001 |
| Region [Non-intensive]: Edge_density_500m | 0.09 | -0.82 – 1.00 | 0.846 |
| Region [Non-intensive]: Cropland_cover_500m | -0.81 | -1.63 – 0.01 | 0.053 |
| Region [Non-intensive]: Tree_cover_500m | -0.33 | -1.13 – 0.48 | 0.426 |
| Random Effects | | | |
| σ2 | 0.24 |  |  |
| τ00 Plot | 0.11 |  |  |
| τ00 Plot_round | 0.6 |  |  |

**Table D.3.9.** Frugivore species richness.

| **Frugivore species richness** | | | |
| --- | --- | --- | --- |
| **Predictors** | **Log-Mean** | **CI** | **p** |
| (Intercept) | 1.53 | 1.27 – 1.79 | <0.001 |
| Edge_density_500m | 0 | -0.21 – 0.22 | 0.979 |
| Cropland_cover_500m | -0.13 | -0.39 – 0.12 | 0.296 |
| Tree_cover_500m | -0.01 | -0.35 – 0.32 | 0.939 |
| Region [Non-intensive] | 0.31 | -0.02 – 0.63 | 0.065 |
| Region [Non-intensive]: Edge_density_500m | 0.05 | -0.44 – 0.54 | 0.83 |
| Region [Non-intensive]: Cropland_cover_500m | 0.07 | -0.29 – 0.42 | 0.71 |
| Region [Non-intensive]: Tree_cover_500m | 0.02 | -0.37 – 0.40 | 0.931 |
| Random Effects | | | |
| σ2 |  |  |  |
| τ00 Plot | 0 |  |  |

**Table D.3.10.** Frugivore abundance.

| **Frugivore abundance** | | | |
| --- | --- | --- | --- |
| **Predictors** | **Log-Mean** | **CI** | **p** |
| (Intercept) | 2.78 | 2.47 – 3.08 | <0.001 |
| Edge_density_500m | -0.09 | -0.35 – 0.17 | 0.51 |
| Cropland_cover_500m | -0.34 | -0.64 – -0.03 | 0.031 |
| Tree_cover_500m | -0.14 | -0.54 – 0.26 | 0.489 |
| Region [Non-intensive] | 0.75 | 0.34 – 1.16 | <0.001 |
| Region [Non-intensive]: Edge_density_500m | 0.25 | -0.45 – 0.96 | 0.481 |
| Region [Non-intensive]: Cropland_cover_500m | 0.45 | -0.02 – 0.92 | 0.06 |
| Region [Non-intensive]: Tree_cover_500m | -0.13 | -0.60 – 0.34 | 0.59 |
| Random Effects | | | |
| σ2 | 0.25 |  |  |
| τ00 Plot | 0.01 |  |  |

**Table D.3.11.** Frugivore Simpson diversity.

| **Frugivore Simpson diversity** | | | |
| --- | --- | --- | --- |
| **Predictors** | **Estimates** | **CI** | **p** |
| (Intercept) | 3.25 | 2.73 – 3.76 | <0.001 |
| Edge_density_500m | -0.11 | -0.53 – 0.30 | 0.587 |
| Cropland_cover_500m | -0.18 | -0.68 – 0.33 | 0.489 |
| Tree_cover_500m | 0.24 | -0.43 – 0.90 | 0.489 |
| Region [Non-intensive] | -0.27 | -0.96 – 0.42 | 0.446 |
| Region [Non-intensive]: Edge_density_500m | 0.32 | -0.81 – 1.45 | 0.582 |
| Region [Non-intensive]: Cropland_cover_500m | -0.15 | -0.92 – 0.63 | 0.711 |
| Region [Non-intensive]: Tree_cover_500m | 0.22 | -0.57 – 1.01 | 0.588 |
| Random Effects | | | |
| σ2 | 0.87 |  |  |
| τ00 Plot | 0 |  |  |

**Table D.3.12.** Frugivore functional diversity (SES.FRic).

| **Frugivore functional diversity (SES.FRic)** | | | |
| --- | --- | --- | --- |
| **Predictors** | **Estimates** | **CI** | **p** |
| (Intercept) | -0.22 | -0.75 – 0.30 | 0.405 |
| Edge_density_500m | 0.22 | -0.25 – 0.70 | 0.355 |
| Cropland_cover_500m | 0.25 | -0.28 – 0.78 | 0.362 |
| Tree_cover_500m | -0.27 | -1.00 – 0.46 | 0.468 |
| Region [Non-intensive] | 0.45 | -0.26 – 1.16 | 0.216 |
| Region [Non-intensive]: Edge_density_500m | -0.29 | -1.46 – 0.88 | 0.623 |
| Region [Non-intensive]: Cropland_cover_500m | -0.7 | -1.49 – 0.10 | 0.087 |
| Region [Non-intensive]: Tree_cover_500m | -0.29 | -1.14 – 0.56 | 0.503 |
| Random Effects | | | |
| σ2 | 0.89 |  |  |
| τ00 Plot | 0 |  |  |

**Table D.3.13.** Frugivore phylogenetic diversity (SES.MPD).

| **Frugivore phylogenetic diversity (SES.MPD)** | | | |
| --- | --- | --- | --- |
| **Predictors** | **Estimates** | **CI** | **p** |
| (Intercept) | 0.16 | -0.45 – 0.77 | 0.612 |
| Edge_density_500m | 0 | -0.49 – 0.50 | 0.989 |
| Cropland_cover_500m | -0.17 | -0.77 – 0.43 | 0.571 |
| Tree_cover_500m | 0.15 | -0.64 – 0.94 | 0.715 |
| Region [Non-intensive] | -0.02 | -0.85 – 0.81 | 0.966 |
| Region [Non-intensive]: Edge_density_500m | -0.04 | -1.39 – 1.31 | 0.954 |
| Region [Non-intensive]: Cropland_cover_500m | 0.55 | -0.37 – 1.47 | 0.239 |
| Region [Non-intensive]: Tree_cover_500m | 0.09 | -0.85 – 1.03 | 0.85 |
| Random Effects | | | |
| σ2 | 1.23 |  |  |
| τ00 Plot | 0 |  |  |

**Table D.3.14.** Abundance of insect-eating phyllostomids.

| **Insect-eating phyllostomid abundance** | | | |
| --- | --- | --- | --- |
| **Predictors** | **Log-Mean** | **CI** | **p** |
| (Intercept) | 0.69 | 0.20 – 1.18 | 0.006 |
| Edge_density_250m | 1.11 | 0.72 – 1.49 | <0.001 |
| Cropland_cover_250m | -0.7 | -1.22 – -0.18 | 0.009 |
| Tree_cover_250m | -0.71 | -1.16 – -0.26 | 0.002 |
| Region [Non-intensive] | 0.55 | -0.10 – 1.19 | 0.095 |
| Region [Non-intensive]: Edge_density_250m | -0.4 | -1.14 – 0.33 | 0.284 |
| Region [Non-intensive]: Cropland_cover_250m | 0.5 | -0.15 – 1.14 | 0.131 |
| Region [Non-intensive]: Tree_cover_250m | 0.72 | -0.05 – 1.49 | 0.065 |
| Random Effects | | | |
| σ2 |  |  |  |
| τ00 Plot | 0 |  |  |

**Table D.3.15.** Abundance of small frugivores.

| **Small frugivore abundance** | | | |
| --- | --- | --- | --- |
| **Predictors** | **Log-Mean** | **CI** | **p** |
| (Intercept) | 2.09 | 1.81 – 2.37 | <0.001 |
| Edge_density_500m | -0.19 | -0.44 – 0.07 | 0.147 |
| Cropland_cover_500m | -0.37 | -0.66 – -0.09 | 0.011 |
| Tree_cover_500m | 0.16 | -0.21 – 0.54 | 0.395 |
| Region [Non-intensive] | 1.15 | 0.80 – 1.50 | <0.001 |
| Region [Non-intensive]: Edge_density_500m | 0.37 | -0.20 – 0.94 | 0.198 |
| Region [Non-intensive]: Cropland_cover_500m | 0.54 | 0.14 – 0.93 | 0.008 |
| Region [Non-intensive]: Tree_cover_500m | -0.41 | -0.84 – 0.01 | 0.055 |
| Random Effects | | | |
| σ2 | 0.19 |  |  |
| τ00 Plot | 0 |  |  |

**Table D.3.16.** Abundance of large frugivores.

| **Large frugivore abundance** | | | |
| --- | --- | --- | --- |
| **Predictors** | **Log-Mean** | **CI** | **p** |
| (Intercept) | 1.85 | 1.48 – 2.22 | <0.001 |
| Edge_density_250m | 0.13 | -0.17 – 0.44 | 0.388 |
| Cropland_cover_250m | -0.25 | -0.62 – 0.12 | 0.191 |
| Tree_cover_250m | -0.42 | -0.81 – -0.03 | 0.034 |
| Region [Non-intensive] | 0.31 | -0.18 – 0.81 | 0.216 |
| Region [Non-intensive]: Edge_density_250m | 0.29 | -0.30 – 0.88 | 0.341 |
| Region [Non-intensive]: Cropland_cover_250m | 0.27 | -0.23 – 0.76 | 0.293 |
| Region [Non-intensive]: Tree_cover_250m | 0.32 | -0.29 – 0.93 | 0.309 |
| Random Effects | | | |
| σ2 |  |  |  |
| τ00 Plot | 0 |  |  |

## **Table D.4.** Results of the Moran´s I test comparing the expected and observed spatial autocorrelation in the residuals of GLMMs analyzing landscape effects on the response variables of aerial insectivores and phyllostomids.

| **Group** | **Model** | **Expected** | **Observed (SD)** | ***p*-value** |
| --- | --- | --- | --- | --- |
| Aerial insectivores | Species richness | -0.037 | -0.032 (0.039) | 0.901 |
|  | Activity | -0.037 | -0.011 (0.039) | 0.5 |
|  | Simpson diversity | -0.037 | -0.016 (0.038) | 0.572 |
|  | Feeding activity (all spp.) | -0.037 | -0.009 (0.036) | 0.437 |
|  | Feeding activity (unserstory spp.) | -0.037 | -0.008 (0.038) | 0.439 |
|  | Feeding activity (canopy spp.) | -0.037 | -0.011 (0.037) | 0.476 |
|  | SES.FRic | -0.037 | -0.022 (0.038) | 0.693 |
|  | SES.MPD | -0.037 | -0.028 (0.036) | 0.814 |
| Phyllostomids | Species richness | -0.067 | -0.012 (0.067) | 0.415 |
|  | Abundance | -0.067 | -0.02 (0.069) | 0.493 |
|  | Simpson diversity | -0.067 | -0.01 (0.067) | 0.403 |
|  | Insect-eating abundance | -0.067 | -0.017 (0.068) | 0.462 |
|  | Small fruit-eating abundance | -0.067 | -0.016 (0.067) | 0.449 |
|  | Large fruit-eating abundance | -0.067 | -0.027 (0.067) | 0.561 |
|  | SES.FRic | -0.067 | -0.017 (0.059) | 0.398 |
|  | SES.MPD | -0.067 | -0.011 (0.064) | 0.385 |

## **Table D.5.1.** Variance inflation factor (VIF) values of the predictors in models analyzing aerial insectivore species richness, activity, Simpson diversity and functional diversity.

| **Response variable** | **Term** | **VIF (95% CI)** |
| --- | --- | --- |
| Aerial insectivore species richness | Edge_density_500m | 3.29 (2.52, 4.45) |
|  | Cropland_500m | 3.09 (2.38, 4.17) |
|  | Tree_cover_500m | 6.74 (4.98, 9.27) |
|  | Region | 2.02 (1.62, 2.68) |
|  | Region:Edge_density_500m | 1.7 (1.39, 2.24) |
|  | Region:Cropland_500m | 2.55 (1.99, 3.42) |
|  | Region:Tree_cover_500m | 3.75 (2.85, 5.1) |
| Aerial insectivore activity | Edge_density_500m | 3.2 (2.47, 4.32) |
|  | Cropland_500m | 3.09 (2.39, 4.16) |
|  | Tree_cover_500m | 6.77 (5.02, 9.28) |
|  | Region | 1.91 (1.54, 2.52) |
|  | Region:Edge_density_500m | 1.74 (1.42, 2.29) |
|  | Region:Cropland_500m | 2.52 (1.98, 3.37) |
|  | Region:Tree_cover_500m | 3.98 (3.02, 5.39) |
| Aerial insectivore Simpson diversity | Edge_density_500m | 3.2 (2.47, 4.32) |
|  | Cropland_500m | 3.1 (2.39, 4.17) |
|  | Tree_cover_500m | 6.77 (5.02, 9.29) |
|  | Region | 1.91 (1.54, 2.52) |
|  | Region:Edge_density_500m | 1.74 (1.42, 2.29) |
|  | Region:Cropland_500m | 2.52 (1.98, 3.37) |
|  | Region:Tree_cover_500m | 3.98 (3.02, 5.4) |
| Aerial insectivore functional diversity (SES.FRic) | Edge_density_500m | 3.23 (2.48, 4.36) |
|  | Cropland_500m | 3.08 (2.37, 4.15) |
|  | Tree_cover_500m | 6.73 (4.97, 9.26) |
|  | Region | 1.93 (1.56, 2.56) |
|  | Region:Edge_density_500m | 1.76 (1.44, 2.33) |
|  | Region:Cropland_500m | 2.55 (1.99, 3.42) |
|  | Region:Tree_cover_500m | 3.95 (2.99, 5.37) |

## **Table D.5.2.** Variance inflation factor (VIF) values of the predictors in models analyzing aerial insectivore phylogenetic diversity and number of feeding buzzes of all aerial insectivores, and understory and canopy species separately.

| **Response variable** | **Term** | **VIF (95% CI)** |
| --- | --- | --- |
| Aerial insectivore phylogenetic diversity (SES.MPD) | Edge_density_250m | 2.82 (2.19, 3.79) |
|  | Cropland_250m | 2.69 (2.1, 3.61) |
|  | Tree_cover_250m | 3.02 (2.33, 4.06) |
|  | Region | 1.33 (1.15, 1.77) |
|  | Region:Edge_density_250m | 2.74 (2.13, 3.68) |
|  | Region:Cropland_250m | 2.59 (2.03, 3.47) |
|  | Region:Tree_cover_250m | 2.56 (2.01, 3.43) |
| Aerial insectivore  feeding buzzes (all spp.) | Edge_density_500m | 3.21 (2.47, 4.32) |
|  | Cropland_500m | 3.11 (2.4, 4.19) |
|  | Tree_cover_500m | 6.68 (4.95, 9.16) |
|  | Region | 1.99 (1.6, 2.63) |
|  | Region:Edge_density_500m | 1.71 (1.41, 2.26) |
|  | Region:Cropland_500m | 2.58 (2.02, 3.45) |
|  | Region:Tree_cover_500m | 3.89 (2.96, 5.27) |
| Aerial insectivore feeding buzzes (unserstory spp.) | Edge_density_500m | 3.26 (2.5, 4.4) |
|  | Cropland_500m | 3.11 (2.39, 4.2) |
|  | Tree_cover_500m | 6.52 (4.83, 8.97) |
|  | Region | 2.19 (1.74, 2.91) |
|  | Region:Edge_density_500m | 1.72 (1.41, 2.27) |
|  | Region:Cropland_500m | 2.67 (2.08, 3.58) |
|  | Region:Tree_cover_500m | 3.73 (2.84, 5.07) |
| Aerial insectivore feeding buzzes (canopy spp.) | Edge_density_500m | 3.2 (2.46, 4.31) |
|  | Cropland_500m | 3.1 (2.39, 4.18) |
|  | Tree_cover_500m | 6.66 (4.94, 9.13) |
|  | Region | 1.98 (1.59, 2.61) |
|  | Region:Edge_density_500m | 1.71 (1.4, 2.25) |
|  | Region:Cropland_500m | 2.57 (2.01, 3.44) |
|  | Region:Tree_cover_500m | 3.87 (2.94, 5.25) |

## **Table D.5.3.** Variance inflation factor (VIF) values of the predictors in models analyzing frugivore species richness, abundance, Simpson diversity and functional diversity.

| **Response variable** | **Term** | **VIF (95% CI)** |
| --- | --- | --- |
| Frugivore species richness | Edge_density_500m | 3.29 (2.39, 4.79) |
|  | Cropland_500m | 4.98 (3.5, 7.33) |
|  | Tree_cover_500m | 9.23 (6.32, 13.74) |
|  | Region | 2.29 (1.73, 3.29) |
|  | Region:Edge_density_500m | 2.04 (1.56, 2.91) |
|  | Region:Cropland_500m | 3.93 (2.81, 5.74) |
|  | Region:Tree_cover_500m | 5.53 (3.87, 8.15) |
| Frugivore abundance | Edge_density_500m | 3.08 (2.26, 4.43) |
|  | Cropland_500m | 4.75 (3.37, 6.93) |
|  | Tree_cover_500m | 8.33 (5.76, 12.28) |
|  | Region | 2.18 (1.67, 3.1) |
|  | Region:Edge_density_500m | 2.12 (1.62, 3.01) |
|  | Region:Cropland_500m | 3.7 (2.67, 5.36) |
|  | Region:Tree_cover_500m | 4.41 (3.15, 6.43) |
| Frugivore Simpson diversity | Edge_density_500m | 3.24 (2.37, 4.67) |
|  | Cropland_500m | 4.81 (3.41, 7.02) |
|  | Tree_cover_500m | 8.35 (5.78, 12.32) |
|  | Region | 2.31 (1.75, 3.29) |
|  | Region:Edge_density_500m | 2.02 (1.56, 2.86) |
|  | Region:Cropland_500m | 3.59 (2.6, 5.19) |
|  | Region:Tree_cover_500m | 4.3 (3.07, 6.26) |
| Frugivore functional diversity (SES.FRic) | Edge_density_500m | 3.24 (2.35, 4.72) |
|  | Cropland_500m | 4.57 (3.23, 6.72) |
|  | Tree_cover_500m | 8.53 (5.85, 12.71) |
|  | Region | 2.15 (1.64, 3.08) |
|  | Region:Edge_density_500m | 2.06 (1.58, 2.95) |
|  | Region:Cropland_500m | 3.62 (2.6, 5.28) |
|  | Region:Tree_cover_500m | 4.79 (3.37, 7.05) |

## **Table D.5.4.** Variance inflation factor (VIF) values of the predictors in models analyzing frugivore phylogenetic diversity and the abundance of insect-eating phyllostomids, and small- and large-sized frugivores.

| **Response variable** | **Term** | **VIF (95% CI)** |
| --- | --- | --- |
| Frugivore phylogenetic diversity (SES.MPD) | Edge_density_500m | 3.22 (2.35, 4.65) |
|  | Cropland_500m | 4.73 (3.36, 6.92) |
|  | Tree_cover_500m | 8.17 (5.65, 12.07) |
|  | Region | 2.29 (1.73, 3.26) |
|  | Region:Edge_density_500m | 2.01 (1.55, 2.86) |
|  | Region:Cropland_500m | 3.57 (2.58, 5.18) |
|  | Region:Tree_cover_500m | 4.3 (3.06, 6.26) |
| Insect-eating phyllostomid abundance | Edge_density_250m | 2.34 (1.77, 3.33) |
|  | Cropland_250m | 3.76 (2.71, 5.45) |
|  | Tree_cover_250m | 3.1 (2.27, 4.46) |
|  | Region | 1.74 (1.38, 2.46) |
|  | Region:Edge_density_250m | 2.08 (1.6, 2.96) |
|  | Region:Cropland_250m | 3.33 (2.43, 4.8) |
|  | Region:Tree_cover_250m | 2.77 (2.05, 3.97) |
| Small frugivore abundance | Edge_density_500m | 3.18 (2.33, 4.58) |
|  | Cropland_500m | 4.94 (3.5, 7.21) |
|  | Tree_cover_500m | 8.95 (6.18, 13.22) |
|  | Region | 2.03 (1.56, 2.88) |
|  | Region:Edge_density_500m | 2.17 (1.66, 3.09) |
|  | Region:Cropland_500m | 4.13 (2.96, 6) |
|  | Region:Tree_cover_500m | 5.67 (3.99, 8.31) |
| Large frugivore abundance | Edge_density_250m | 2.4 (1.81, 3.42) |
|  | Cropland_250m | 3.08 (2.26, 4.44) |
|  | Tree_cover_250m | 4.07 (2.92, 5.92) |
|  | Region | 1.66 (1.32, 2.34) |
|  | Region:Edge_density_250m | 2.34 (1.77, 3.34) |
|  | Region:Cropland_250m | 2.62 (1.95, 3.74) |
|  | Region:Tree_cover_250m | 3.18 (2.33, 4.59) |

## **Table D.6.** Results of Wilcoxon signed rank tests comparing model predictions of response variables under current and future landscape scenarios. Significant deviations from the current scenario are marked in **bold**.

| **Group** | **Response** | **Region** | **Scenario** | **∆Median (95% CI)** | **p-value** |
| --- | --- | --- | --- | --- | --- |
| Aerial insectivores | Activity | Non-intensive | Deforestation | **107.46 (70.04, 172.25)** | **0.0001** |
|  |  |  | Reforestation | **-109.14 (-458.37, -38.39)** | **0.0004** |
|  | SES.FRic | Non-intensive | Deforestation | **-0.98 (-1.57, -0.46)** | **0.0009** |
|  |  |  | Reforestation | -0.11 (-0.59, 0.38) | 0.7609 |
|  | SES.MPD | Non-intensive | Deforestation | **0.88 (0.38, 1.51)** | **0.0004** |
|  |  |  | Reforestation | **-0.6 (-0.97, -0.32)** | **0.0001** |
|  | Feeding buzzes | Non-intensive | Deforestation | **2.83 (1.16, 4.66)** | **0.0031** |
|  |  |  | Reforestation | **-10.17 (-18.64, -5.58)** | **0.0001** |
|  | Feeding buzzes understory | Non-intensive | Deforestation | 0.13 (-0.4, 0.43) | 0.3910 |
|  |  |  | Reforestation | **-0.75 (-1.86, -0.43)** | **0.0001** |
| Phyllostomids | Abundance | Intensive | Deforestation | **0.48 (-1.46, 2.58)** | **0.4609** |
|  |  |  | Reforestation | **-3.42 (-6.42, -2.21)** | **0.0078** |
|  |  | Non-intensive | Deforestation | **-11.14 (-19.7, -5.2)** | **0.0078** |
|  |  |  | Reforestation | **9.04 (4.57, 13)** | **0.0078** |
|  | Simpson diversity | Non-intensive | Deforestation | **1.07 (0.45, 1.63)** | **0.0078** |
|  |  |  | Reforestation | -0.22 (-0.81, 0.05) | 0.0781 |
|  | Frugivore SES.FRic | Non-intensive | Deforestation | -0.17 (-0.53, 0.03) | 0.2500 |
|  |  |  | Reforestation | **-0.09 (-0.16, -0.01)** | **0.0391** |
|  | Insect-eating abundance | Intensive | Deforestation | **1.73 (0.28, 10.24)** | **0.0391** |
|  |  |  | Reforestation | 0.48 (-5.87, 8.15) | 1.0000 |
|  |  | Non-intensive | Deforestation | 1.24 (-0.81, 3.16) | 0.1484 |
|  |  |  | Reforestation | **1.63 (0.62, 2.72)** | **0.0156** |
|  | Small fruit-eating abundance | Intensive | Deforestation | 1.41 (0.14, 3) | 0.0547 |
|  |  |  | Reforestation | **-2.28 (-4.66, -0.81)** | **0.0078** |
|  |  | Non-intensive | Deforestation | **-8.51 (-16.09, -3.71)** | **0.0078** |
|  |  |  | Reforestation | **7.13 (3.55, 10.92)** | **0.0078** |
|  | Large fruit-eating abundance | Intensive | Deforestation | 0.01 (-1.89, 1.85) | 1.0000 |
|  |  |  | Reforestation | 0.49 (-0.67, 2.3) | 0.3125 |


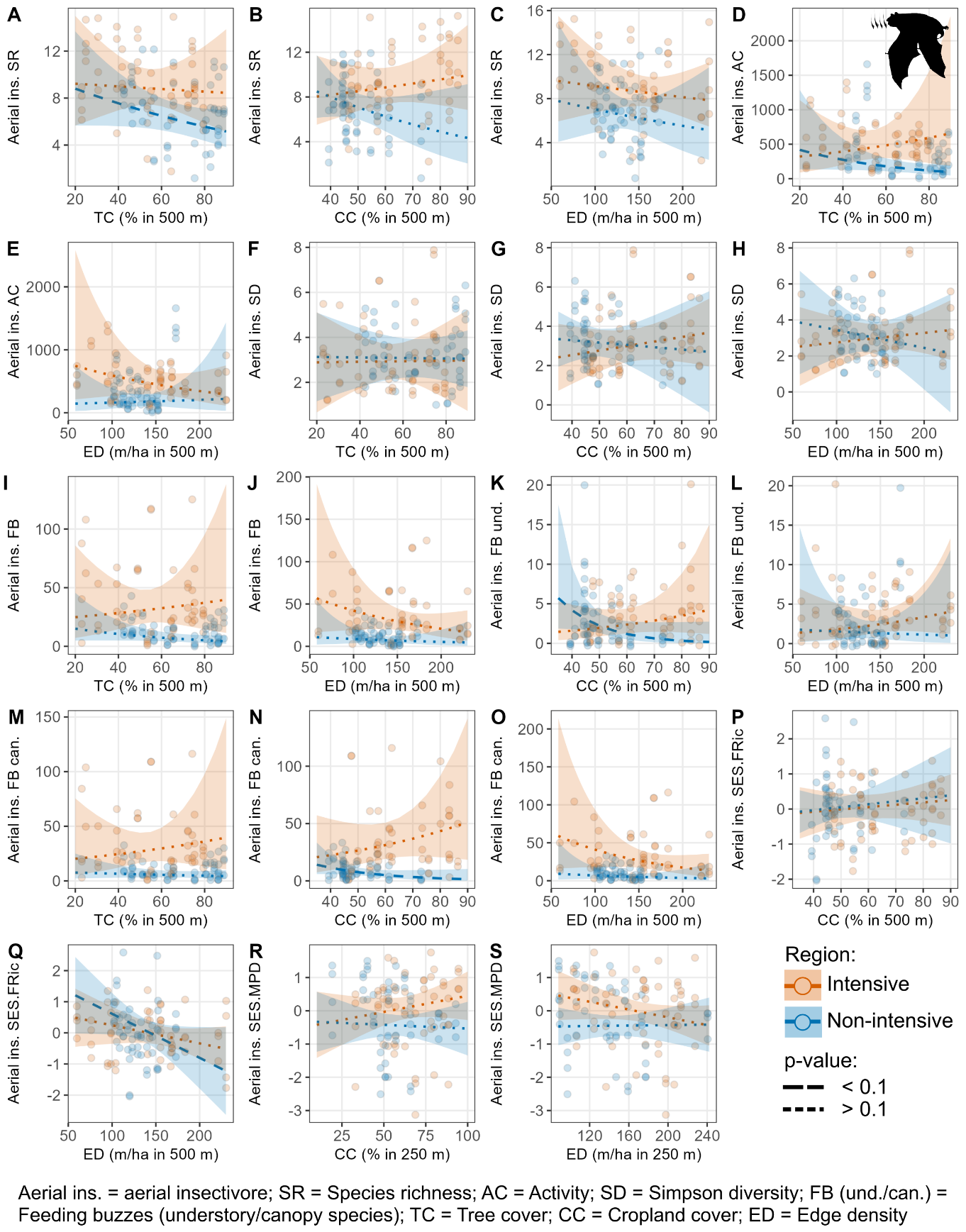


**Figure D.1.** Non-significant effects of landscape predictors on aerial insectivore response variables. Orange lines and CIs represent trends in the intensive region, blue lines and CIs represent trends in the non-intensive region. Points represent the raw values. Marginally significant trends (0.1 > p > 0.05) are represented by dashed lines.


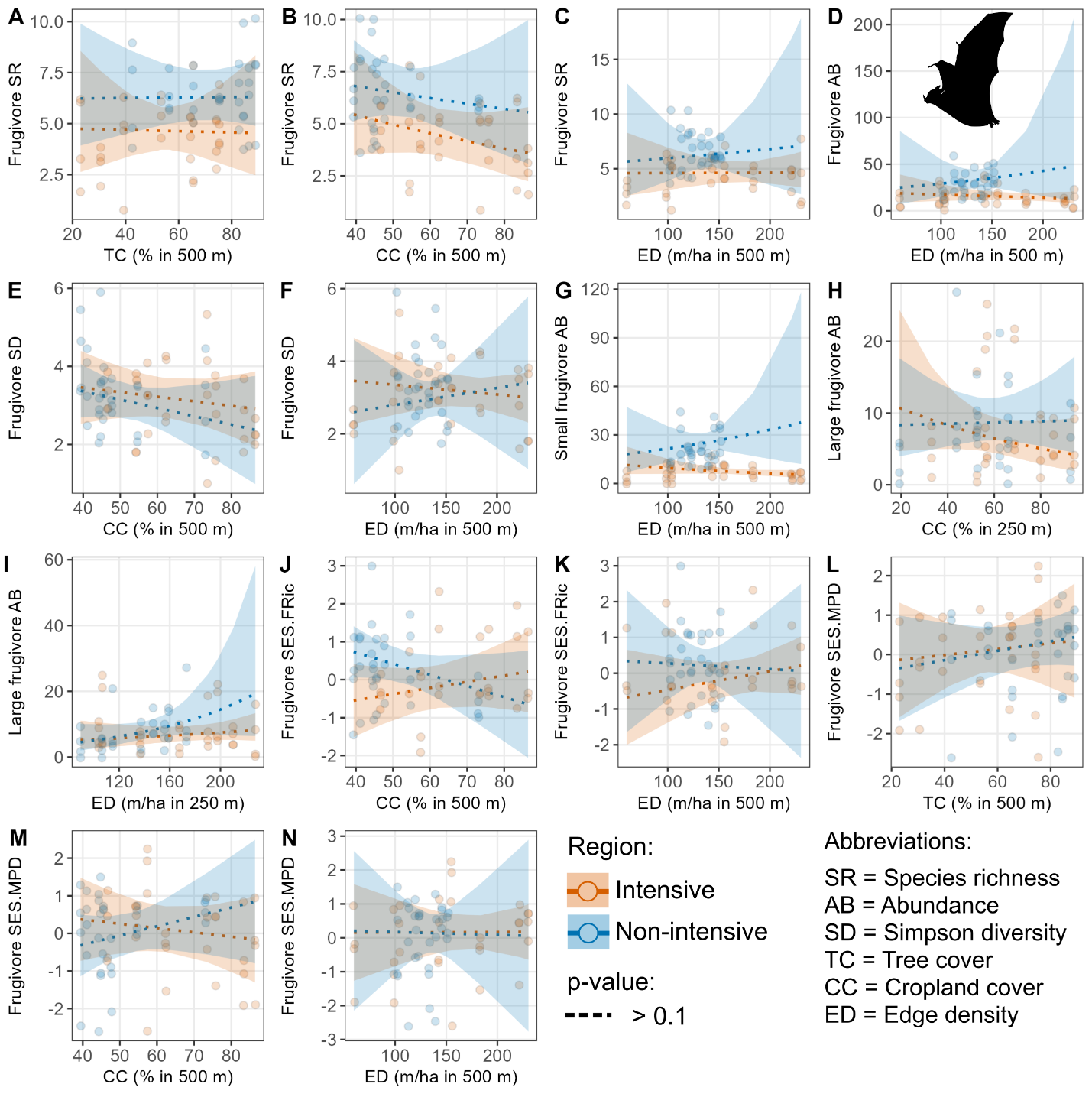


**Figure D.2.** Non-significant effects of landscape predictors on frugivore and insect-eating phyllostomid response variables. Orange lines and 95% CIs represent trends in the intensive region, while blue lines and CIs represent the non-intensive region. Points represent the raw values in each region.
